# Understanding the evolution of lithium dendrites at Li_6.25_Al_0.25_La_3_Zr_2_O_12_ grain boundaries via operando microscopy techniques

**DOI:** 10.1038/s41467-023-36792-7

**Published:** 2023-03-09

**Authors:** Chao Zhu, Till Fuchs, Stefan A. L. Weber, Felix. H. Richter, Gunnar Glasser, Franjo Weber, Hans-Jürgen Butt, Jürgen Janek, Rüdiger Berger

**Affiliations:** 1 Max Planck Institute for Polymer Research, Ackermannweg 10, 55128 Mainz, Germany; 2 Institute of Physical Chemistry & Center for Materials Research, Justus Liebig University Giessen, Heinrich-Buff Ring 17, 35392 Giessen, Germany; 3 Institute of Physics, Johannes Gutenberg University Mainz, Staudingerweg 7, 55128 Mainz, Germany

**Keywords:** Batteries, Electrochemistry, Solid-state chemistry, Microscopy, Energy storage

## Abstract

The growth of lithium dendrites in inorganic solid electrolytes is an essential drawback that hinders the development of reliable all-solid-state lithium metal batteries. Generally, ex situ post mortem measurements of battery components show the presence of lithium dendrites at the grain boundaries of the solid electrolyte. However, the role of grain boundaries in the nucleation and dendritic growth of metallic lithium is not yet fully understood. Here, to shed light on these crucial aspects, we report the use of operando Kelvin probe force microscopy measurements to map locally time-dependent electric potential changes in the Li_6.25_Al_0.25_La_3_Zr_2_O_12_ garnet-type solid electrolyte. We find that the Galvani potential drops at grain boundaries near the lithium metal electrode during plating as a response to the preferential accumulation of electrons. Time-resolved electrostatic force microscopy measurements and quantitative analyses of lithium metal formed at the grain boundaries under electron beam irradiation support this finding. Based on these results, we propose a mechanistic model to explain the preferential growth of lithium dendrites at grain boundaries and their penetration in inorganic solid electrolytes.

## Abstract (web summary)

Lithium metal penetration into solid-state electrolytes is a major drawback for developing all-solid-state batteries. Here, authors, via operando force microscopy, demonstrate that the trapping of electrons in garnet-type solid-state electrolyte grain boundaries is the origin of cell failure.

## Introduction

Garnet-type solid electrolytes are considered attractive components for use as ceramic separator material in solid-state batteries (SSB), which may be next-generation energy storage devices^1–4^. In practice, such ceramic separators are polycrystalline and thus they contain various types of grain boundaries^2,3^. These grain boundaries, which are the transition regions between crystalline grains of different orientation, generally have different properties than the grains themselves and cause microstructural and electrochemical heterogeneities^5,6^. This has been proved to be a critical issue for the stable operation of the lithium metal anode—which is a key target in SSB development. Many researchers have observed that lithium dendrites tend to spread in grain boundaries of garnet-type solid electrolytes^7–10^ such as Li_7_La_3_Zr_2_O_12_. However, there are no physical spaces or voids at grain boundaries available for lithium penetration^11–13^. Garnet-type solid electrolytes form a stable interphase in contact with lithium metal^14^, have a wider electrochemical stability window^15^, and greater mechanical strength^16^ than other types of solid electrolytes. However, lithium dendrite penetration into garnet-type solid electrolytes still limits further application. Ex situ observations^11^, density functional theory (DFT) or physical field-based simulations^17–20^ and microscopic electrochemical measurement methods^21,22^ have been used to characterize lithium-ion transport, lithium-dendrite nucleation, and lithium-dendrite growth at grain boundaries in garnet-type solid electrolytes. Unfortunately, the conclusions drawn from those experiments are contradictory, and the role that grain boundaries played in lithium-dendrite nucleation and growth is not fully understood^23,24^. Lu et al. reported that grain boundaries have low lithium ion conductivity^25^, which blocks ion transport and leads to the formation of hot spots for lithium-dendrite nucleation in LLZO due to a local variation of current density. In contrast, Cheng et al. reported that grain boundaries have a higher ionic conductivity than bulk grains^21^. Moreover, Song et al. reported that a higher electronic conductivity at grain boundaries compared to that of bulk grains is responsible for lithium dendrite nucleation at grain boundaries^26^. In summary, it is not clear whether high or low ionic conductivity or even the electronic properties at grain boundaries play the determining role in lithium nucleation. Furthermore, it is unclear how dendritic growth is subsequently promoted. In order to address both issues, operando methods are beneficial.

Recently, researchers investigated the electronic band structure of grain boundaries with high spatial resolution spectroscopy^27^. They found that half of the grain boundaries exhibited a smaller band gap. Grain boundaries with small band gap may serve as channels for an electron leakage current. This finding hints that electron leakage currents in devices might play a role for dendritic growth. However, electron microscopy methods typically cannot be used to study nano-scale electrical processes in an operating device, i.e. during battery charging or discharging. Here, techniques featuring high spatial resolution characterization under practical battery operation conditions are beneficial for understanding the dynamics of preferential lithium nucleation and growth in grain boundaries. In recent years, high-resolution Kelvin probe force microscopy (KPFM) and time-resolved electrostatic force microscopy (tr-EFM) based on scanning force microscopy (SFM) were developed. These microscopy techniques can track potential changes with nanometer-scale lateral resolution and compare ion mobility with microsecond-scale temporal resolution^28–30^. Both techniques operated in an inert atmosphere hold the key to understanding the dynamics of preferential lithium penetration in grain boundaries.

To clarify the role of different ionic or electronic conduction properties for lithium dendrites that more likely grow along grain boundaries, we used cubic phase Li_6.25_Al_0.25_La_3_Zr_2_O_12_ (LLZO) as a model solid electrolyte. Li_6.25_Al_0.25_La_3_Zr_2_O_12_ is widely accepted as representative material for LLZO-based garnet-type solid electrolyte while pure Li_7_La_3_Zr_2_O_12_ is typically not cubic and not well ion-conducting^31^. We then prepared symmetric Li|LLZO|Li cells to investigate their nanometer-scale local electronic properties. We found a local Galvani potential (the Galvani potential is the inner electrostatic potential, relative to a reference state *ϕ* = 0 at infinite distance from the surface of the sample) drop at grain boundaries in LLZO near the lithium counter electrode (Li-CE, plating side) during lithium plating. With complementary measurements performed with tr-EFM and electron-beam injection, we associate this time-dependent potential drop to the preferential accumulation of electrons at grain boundaries near the plating side, which allows lithium ions to be reduced locally. These methods also were used to investigate amorphous Li_3_PO_4_ as a second type of solid electrolyte that does not have grain boundaries, which showed that those methods are beneficial for a wide range of solid electrolytes. This further verifies the reliability of our conclusions on the specific conduction properties of LLZO grain boundaries. Based on these findings, our model explains the favored deposition of lithium metal in grain boundaries of garnet-type solid electrolytes and subsequent dendrite growth.

## Results and discussion

### Operando KPFM measurements

**Fig. 1:**
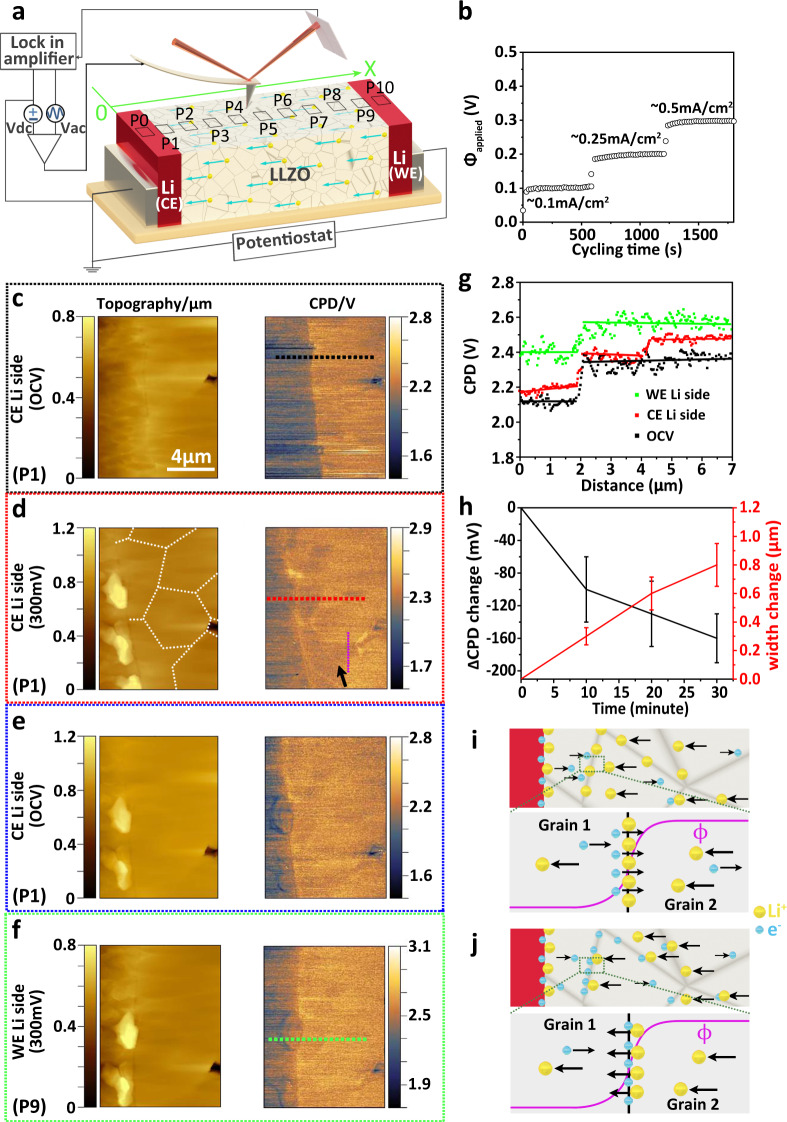
Operando KPFM measurements on LLZO close to electrode. **a** Schematic of KPFM on the LLZO surface of a symmetric Li|LLZO|Li cell. The cell was cycled via a potentiostat. **b** Cycling performance of the symmetric Li|LLZO|Li cell with potentials of 100, 200, and 300 mV applied to the Li-WE at the set constant current densities of 0.1 mA/cm^2^, 0.25 mA/cm^2^, and 0.5 mA/cm^2^. **c**–**f** Topography images and the corresponding CPD maps of OCV state, lithium plating at the Li-CE, relaxed and re-equilibrated state, and lithium stripping state at Li-WE with a potential difference of 300 mV between the two electrodes. **g** Line profiles extracted from CPD maps in **c** (dashed black line), **d** (dashed red line), and **f** (dashed green line). **h** Measured ∆CPD and width change as a function of time under 300 mV for the region along dotted purple line in CPD maps in **d**. The error bars correspond to statistics at different data points along dotted purple line. **i**, **j** are two hypothetical scenarios for the formation of a space charge layer at grain boundaries. Scenario **i** results from different conduction properties of lithium ions. In this scenario, the Galvani potential would drop at all the grain boundaries across the whole LLZO pellet. Scenario **j** results from different conduction properties of electrons at grain boundaries, respectively.

X-ray diffraction measurements were carried out to ensure cubic phase purity of LLZO (Supplementary Fig. 1). Analyses of the electrochemical impedance spectroscopy (EIS) measurements show that grain boundaries in LLZO add resistance to the ceramic material (Supplementary Fig. 2 and Supplementary Table 1). To probe variations of the local electric properties of LLZO, we performed operando KPFM measurements at different positions along the LLZO surface of a symmetric Li|LLZO|Li cell (P0 to P10 in Fig. 1a). The KPFM measurements were performed first at open circuit voltage (OCV) and then with a constant current density of 0.1 mA cm^−2^, 0.25 mA cm^−2^ and 0.5 mA cm^−2^ applied to the cell, respectively. Lithium was then stripped from the lithium working electrode (Li-WE) and plated at the Li-CE. The corresponding potential applied to the Li-WE (*ϕ*_applied_) relative to the grounded Li-CE was almost constant (Fig. 1b). Fracture surfaces of electrolyte pellets exhibited a rough surface thus causing a crosstalk with the measured contact potential difference (CPD) signal between SFM tip and sample by KPFM^32^. The latter reduced the lateral resolution. Therefore, we polished the surfaces by argon-ion milling (Supplementary Figs. 3 and 4). The average CPD value of a freshly broken LLZO and an argon-ion milled surface did not differ (Supplementary Fig. 5). Thus, the surface properties of LLZO were not altered by polishing (Supplementary Note 1).

KPFM measurements at OCV conditions: At OCV, the topography of the Li-CE|LLZO interface (P1 in Fig. 1a) exhibits a contrast due to different etching rates during argon-ion polishing, which allows us to distinguish the Li-CE from the LLZO. Even more clearly, the two materials can be distinguished in the measured CPD image (Fig. 1c). The Li-CE features a lower CPD value (≈2.1 V) compared to LLZO (≈2.35 V).

KPFM measurements under current load — plating side (CE): a constant current density of ~0.5 mA cm^−2^ applied to the symmetric Li|LLZO|Li cell leads to an increase of the voltage difference of 300 mV, which caused lithium stripping from the Li-WE and plating at the Li-CE. The volume of the Li-CE increased, manifested by a change in topography adjacent to the LLZO interface. On top of the Li-CE we measured protrusions with a height of 300 nm for a plating time of 30 min (Fig. 1d). Even though the Li-CE was grounded, the measured CPD value increased slightly by 50–80 mV. This increase in CPD scales with the magnitude of the applied potential and was observed only on the Li-CE surface near the Li-CE|LLZO interface. We attribute this slight increase to the presence of a thin lithium-ion conducting passivation layer on the lithium metal cross-section^33^, which formed after breaking the Li|LLZO|Li cell and subsequently polishing it, owing to the high reactivity of lithium metal (Supplementary Figs. 6–8 and Supplementary Note 2).

On the LLZO surface near the interface with the Li-CE, the measured CPD values are 80–100 mV lower in some areas than in neighboring regions after a plating time of 30 min (dark arrow in Fig. 1d). These areas are distributed unevenly and are located at the Li-CE|LLZO interface, but are also found a few micrometers away from the interface. Based on the shape of defects measured in topography and the CPD map of the same LLZO region in the OCV state (Supplementary Fig. 9), we attribute these low equipotential areas to grain boundaries (dotted white line in Fig. 1d).

In addition, we observe a very light bright contrast at very few grain boundaries in the CPD map (Fig. 1d) that can indicate locally a slightly lower work function. We speculate that a bipolar polarization effect leads to reversible chemical polarization at grain boundaries that are crossed by the ionic current. As proven first by Reitz et al.^34^ and later demonstrated for lithium-based systems by Liu et al.^35^, local electronically conducting regions can shortcut the ionic current and lead to chemical polarization. This is a local effect within the grain boundary, which explains only the local very light bright contrast. However, it does not explain the key observation, which is the step-like CPD signal on most grain boundaries close to Li-CE (Fig. 1d).

There are two possible explanations for the step-like decrease in CPD for some grains compared with other grains with increasing plating time. On the one hand, a lower CPD value for some grains may indicate their change of work function due to an irreversible decomposition or a lithium reduction in those grains. In that case, ∆CPD would persist after switching *ϕ*_applied_ back to 0. On the other hand, a lower CPD of those particular grains may indicate a drop of the local Galvani potential at grain boundaries to neighboring grains. The drop of CPD indicates a drop of the Galvani potential, as evidenced by additional measurements on Li|LLZO|Au Hebb-Wagner cells (Supplementary Figs. 10–12 and Supplementary Note 3). In that case, ∆CPD would be reversible and return to 0 after *ϕ*_applied_ is switched back to 0. To check whether these changes in CPD at grain boundaries were transient or permanent, we set the external current to 0 (*ϕ*_applied_ = 0). Now, the CPD map (Fig. 1e) again resembles the one measured initially at OCV (Fig. 1c). However, the lithium protrusions owing to plated lithium on top of the lithium electrode remained. Therefore, we attribute the ∆CPD for *ϕ*_applied_ > 0 to a drop of the Galvani potential at grain boundaries near the Li-CE|LLZO interface.

KPFM measurements under current load — stripping side (WE): To investigate the Li-WE side (P9 in Fig. 1a), we kept the SFM tip at the same scan area but switched the connectors of the potentiostat. This turned the original Li-WE into the grounded Li-CE and the former Li-CE into the new Li-WE. Upon applying an external current equivalent to an applied potential of 300 mV, we found that the CPD on top of the Li-WE increased by 300 mV. Under this condition, lithium ions are now stripped from the new Li-WE. The CPD value of the LLZO near the Li-WE|LLZO interface increased by approximately 200 mV (Fig. 1f). This increase was less than the 300 mV of the externally applied potential to the Li|LLZO|Li cell. We attribute the difference in potential to a high interface resistance of Li|LLZO due to a degrading physical contact for an extended cycling duration^36,37^. More importantly, close to the Li-WE side, we observed no drop of Galvani potential at grain boundaries (green curve in Fig. 1g along the green dashed line of Fig. 1f). As mentioned above, a drop of the Galvani potential is only observed at grain boundaries close to Li-CE (red curve in Fig. 1g along the red dashed line of Fig. 1d). Furthermore, we measured the CPD difference $\triangle {{{{{\rm{CPD}}}}}}={{{{{{\rm{CPD}}}}}}}_{{{{\rm{low}}}}\,{{{\rm{CPD}}}}\,{{{\rm{area}}}}}\; -{{{{{{\rm{CPD}}}}}}}_{{{{\rm{high}}}}\,{{{\rm{CPD}}}}\,{{{\rm{area}}}}}$ along the dotted purple line in Fig. 1d, which decreases with plating time (Fig. 1h). This means the Galvani potential drops at grain boundaries close to Li-CE increases with plating time. In addition, the width of the low equipotential areas along grain boundaries in the CPD maps increases continuously (Fig. 1h and Supplementary Fig. 13).

KPFM measurements under current load — grain boundaries: If there is a uniform LLZO bulk resistance, the gradient of the Galvani potential, $\partial \phi /\partial x$, in LLZO will be constant across the LLZO for *ϕ*_applied_ > 0. Thus, the measured CPD will decrease linearly in LLZO from the Li-WE to the Li-CE. Therefore, the measured CPD drop at grain boundaries close to Li-CE indicates the presence of a space charge layer. Two scenarios could be the origin of a space charge layer at grain boundaries near the Li-CE at which lithium is being plated: The first scenario is that grain boundaries act as a potential barrier for lithium ions owing to different transport properties. The presence of a barrier causes lithium ions to accumulate close to grain boundaries. Local charge neutrality is then achieved by electrons at grain boundaries. In this case, lithium ions and electrons form a space charge layer, which then partially shields the external electric potential (Fig. 1i). However, in this situation, Galvani potential drops at grain boundaries should be independent from the position of the grain boundary, and should not only be observed close to the Li-CE. The second scenario is that the formation of a space charge layer results from different electronic conduction properties at the grain boundaries compared to in-grain parts (Fig. 1j). Even though we regard LLZO as a pure ionic conductor (≈10^−4^ S cm^−1^ at 25 °C), it also exhibits a low electronic conductivity of ≈10^−7^ − 10^−11^ S cm^−1^^31,38^.

**Fig. 2:**
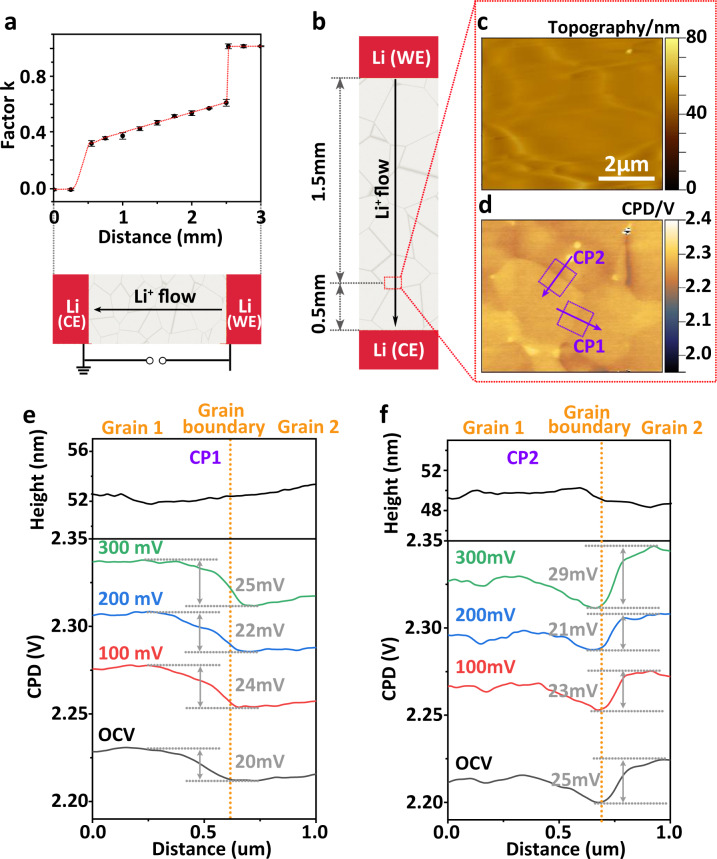
Operando KPFM measurements on LLZO far from electrode. **a** The factor *k* calculated  at different points on Li|LLZO|Li surfaces at 300 mV potential difference between the two electrodes. The error bars corresponds to three independent experiments. **b** Schematic diagram of the position of the KPFM measurement on the LLZO surface. **c**, **d** Topography and CPD maps (6 µm × 5 µm) of the regions within the dotted box in **b** in an OCV state. **e**, **f** Averaged line profiles of the topography and CPD extracted from areas CP1 and CP2 in the direction of the arrow under different potentials applied to the WE, respectively.

KPFM measurements under current load—  LLZO surface far from lithium electrodes: To further prove which scenario explains the observed Galvani potential drop at grain boundaries close to Li-CE, we performed KPFM measurements at other areas (P0, P2–P8, P10 as indicated in Fig. 1a) under an applied potential of 300 mV to the WE in the symmetric cell Li|LLZO|Li. Then we plotted the factor *k*(x)

1 $$k\left(x\right)=\frac{{{CPD}\left(x\right)}_{{{{{{\rm{applied}}}}}}}-{{CPD}\left(x\right)}_{{{{{{\rm{OCV}}}}}}}}{{\phi }_{{{{{{\rm{applied}}}}}}}},$$

where *x* is the distance between a LLZO surface point and the Li-CE, ${{CPD}\left(x\right)}_{{{{{{\rm{OCV}}}}}}}$ is the CPD value of the LLZO surface point in the OCV state. ${{CPD}\left(x\right)}_{{{{{{\rm{applied}}}}}}}$ is the CPD value of the LLZO surface point with an applied potential ${\phi }_{{{{{{\rm{applied}}}}}}}$ on cell (Fig. 2a). The plot indicates distinct areas: the two interfaces of the lithium electrodes where *k* changes by 0.3–0.4. These step-like changes at both Li|LLZO interfaces indicate a higher interface resistance due to a poor physical contact^36,39–41^. The inner part of LLZO far from the interfaces exhibits a change of *k* from ≈0.3 to ≈0.6 at a constant slope of ≈0.15 mm^−1^. A constant slope of *k* is also detected under different applied potentials (Supplementary Fig. 14), which indicates that a constant electric field acts along the bulk LLZO. Such a constant slope of *k* was also observed for amorphous Li_3_PO_4_. We used amorphous Li_3_PO_4_ as a second type of solid electrolyte (ionic conductivity is around 10^−7 ^S cm^−1^ at 25 °C), that does not contain any grain boundaries and should be spatially homogenous. In particular, we did not observe a sudden drop in Galvani potential in Li_3_PO_4_ close to Li-CE under an applied potential to Li-WE in the symmetric cell Li|Li_3_PO_4_|Li (Supplementary Fig. 15 and Supplementary Note 4).

In order to study grain boundaries far from both Li-CE and Li-WE in more detail, we performed KPFM measurement at position P3 (Fig. 1a), which is 0.5 mm from the Li-CE and 1.5 mm from Li-WE (Fig. 2b). The topography image showed no voids or defects (Fig. 2c). The corresponding CPD map shows a difference between in-grain parts and grain boundaries (Fig. 2d). Average line profiles in topography and CPD along the direction of the arrow in the two regions CP1 and CP2 (purple rectangular wireframe) show no correlation between the topography and the measured CPD (Fig. 2e, f). Both CP1 and CP2 contain two different grains and one grain boundary, respectively. Different grains show CPD differences of several tens of millivolts. We attribute this difference to the different crystal orientations or local impurities^42,43^. Next, we applied different constant external current densities to the Li|LLZO|Li cell, resulting in a different potential *ϕ*_applied_ applied to the WE. Now the average CPD surface profiles (CP1 and CP2), which are always recorded at the same position, shifted by approximately 50, 85, and 112 mV at a corresponding *ϕ*_applied_ of 100, 200, and 300 mV, respectively (Fig. 2e, f, Supplementary Fig. 16). In particular, those CPD differences between two grains in CP1 are approximately 24, 22 and 25 mV at 100, 200 and 300 mV, respectively. Thus, the CPD difference between the two grains remains constant and unaffected by *ϕ*_applied_. The second CPD line profile CP2 follows a profile that is almost perpendicular to CP1. A potential directional dependence is  excluded in this way. Thus, there is no evident drop of electric potential at grain boundaries in this region and consequently no ion-barrier.

In summary, KPFM measurements show that drops in Galvani potential take place only at grain boundaries near the Li-CE, where lithium is being plated. Thus, grain boundaries do not have different ionic transport properties. Consequently, a scenario sketched in Fig. 1i is unlikely. This conclusion is further supported by tr-EFM, which are discussed in the next section. The latter finding indicates that we need to consider different electron transport properties in grain boundaries compared to the grain bulk (Fig. 1j).

### Time-resolved EFM (tr-EFM)

**Fig. 3:**
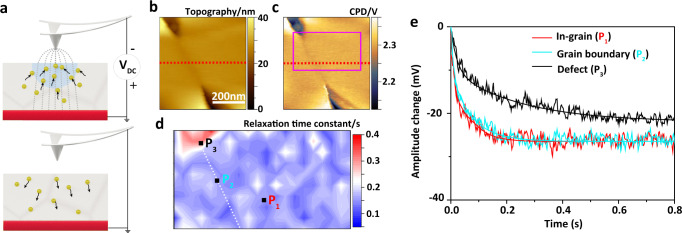
Tr-EFM measurement on LLZO surface. **a** Schematic illustration of the principle of tr-EFM on LLZO. **b** SFM topography of LLZO including voids, grain boundaries, and in-grain parts. The RMS roughness is 4.1 nm at 500 nm × 500 nm. **c** CPD map of the same place as in **b**. **d** Lithium ion relaxation time constant (τ) map of the purple area in **c**. **e** Amplitude change vs time curve of three points (in-grain, grain boundary, and defect void) after removing the −3 V DC voltage from the SFM tip.

In tr-EFM, lithium ions are moved by the electric field emanating from the SFM tip due to an additional direct current (DC) voltage. Once the DC voltage is switched off, the additional electric field of the tip is removed and the mobile ions return back into an equilibrium state (Fig. 3a). The tr-EFM method has previously been used to investigate time-resolved ion dynamics in ion-conducting materials^44–46^. Here, we compare lithium ion relaxation processes between grain boundaries and in-grain parts^47,48^.

For tr-EFM, we chose an area on the LLZO surface within 500 nm distance from the Li-CE that contains different grains, grain boundaries and voids (Fig. 3b). A horizontal cross-section (dotted red line in Fig. 3b) reveals that the grain boundary region has a width of 30–40 nm and is approximately 2 nm lower than in-grain parts (Supplementary Fig. 17). KPFM results show that the grain boundary has an about 40 mV lower CPD value than in-grain areas (Fig. 3c and Supplementary Fig. 17). The voids show CPD values some 100 to 200 mV lower than in-grain areas. We attribute these lower CPD values to a Li_2_CO_3_ layer that formed in voids during prolonged storage of LLZO samples or during the sintering process (Supplementary Fig. 18).

The tr-EFM measurement was performed in an area of 400 nm × 200 nm marked by a purple rectangle in Fig. 3c. This area was uniformly divided into 20 × 10 pixels. At each pixel, we fitted the SFM tip amplitude change at the secondary resonance frequency vs. time with a stretched-exponential function to obtain the lithium-ion relaxation time constant *τ* (Fig. 3d) (more details in the Methods section). The void corresponds to *τ* values of (296 ± 40) ms, and all other areas correspond to values of (166 ± 20) ms. In particular, there is no difference between the grain boundary and the in-grain area (Fig. 3e). For example, the amplitude shift vs time dependence of three points at the in-grain region P_1_, the grain boundary P_2_ and the defect/void P_3_ are plotted in Fig. 3e. The changes in measured amplitude as a function of time overlap at P_1_ and P_2_. Thus, we conclude that grain boundaries near the Li-CE and the in-grain areas in LLZO show similar ionic conductivity. In other words, grain boundaries do not provide faster or slower ion conduction channels in the LLZO solid electrolyte investigated here (i.e., Li_6.25_Al_0.25_La_3_Zr_2_O_12_). However, the amplitude changes vs time  are slower at voids, which are partly filled with Li_2_CO_3_ (e.g., P_3_ in Fig. 3e). A higher time constant at void regions is reasonable, owing to the presence of Li_2_CO_3_, which is known to have a lower ionic diffusivity than that of pure LLZO^12,49,50^.

### Electron-beam injection

The above tr-EFM experiments indicate that there is a negligible difference in ion mobility at grain boundaries compared to the in-grain parts even at the grain boundaries close to Li-CE. Thus, grain boundaries do not represent an additional barrier for lithium ions. Therefore, the electric potential drop at grain boundaries in KPFM measurements near the Li-CE interface might result from differences in electron transport properties in grain boundaries (Fig. 1j). However, garnet-type electrolytes are ion conductors with a very poor electron conductivity of 10^−8^–10^−11^ S cm^−1^ at 25 °C^31,38,51^, which is about 10^4^–10^7^ times lower than their ionic conductivity at the same temperature. Therefore, it is challenging to quantify the electronic properties of LLZO using standard methods, i.e., by measuring the electronic conductivity in cells with an ion-blocking electrode (Hebb-Wagner cell)^31^. Recently, a method based on electron beam-induced alkali metal growth in different kinds of ionic conductors was reported^52,53^. This method was used to investigate the effect of local impurities on lithium metal nucleation and growth from garnet-type solid electrolyte-induced by electrons^52^.

**Fig. 4:**
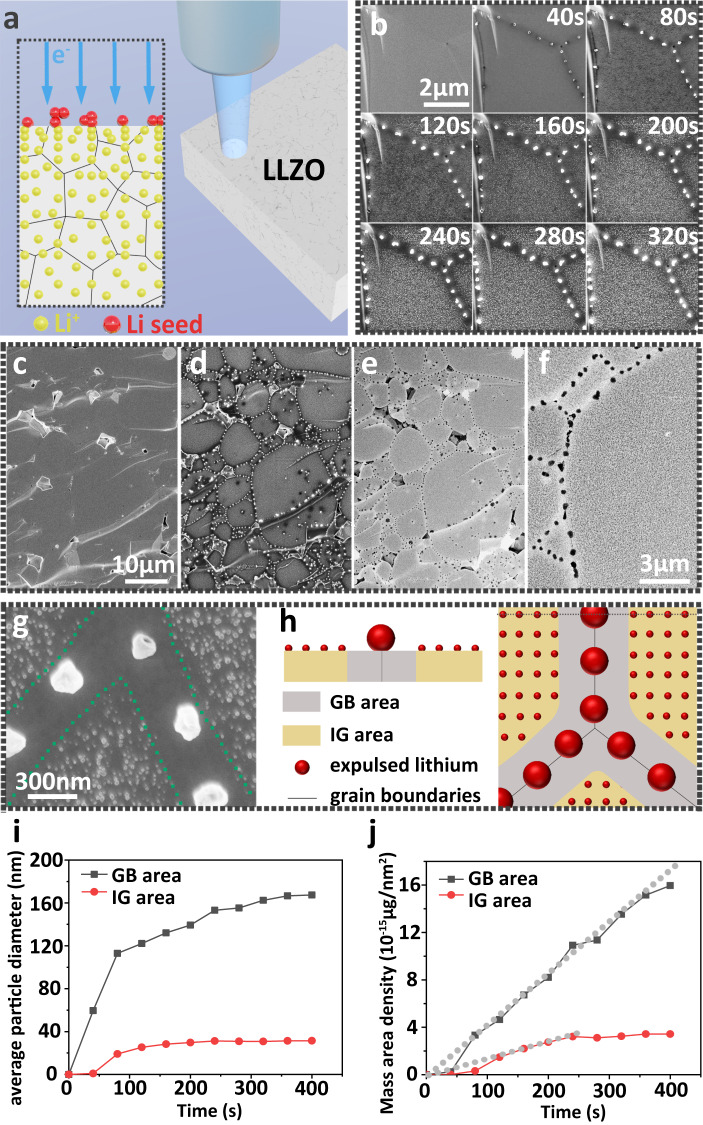
Electron beam irradiation of LLZO surfaces. **a** Schematic diagram of electron injection causing a reduction of lithium ions in LLZO into lithium metal. **b** Morphological evolution of lithium particles on a LLZO cross-section with increasing duration under electron-beam irradiation. The surface includs grain boundaries and in-grain areas. **c** Initial LLZO cross-section morphology under electron-beam irradiation on a large scale. **d** Morphology changes after electron-beam irradiation for 400 s. **c** and **d** are recorded by measuring  in secondary electron detection mode. **e** Same area as in **d**, but electrons were collected with a backscattering electron detector. **f** Partial enlarged view of **e**. **g** Enlarged view of lithium expulsion at inner-grain (IG) area and grain boundary (GB) area. The IG area has only tiny lithium expulsion particles, whereas the GB area exhibits  adjacent regions with no expulsions. The green dotted lines are the boundaries of IG area and GB area. **h** Schematic of different lithium expulsions at IG area and GB area**. i** Average diameter and **j** mass areal density evolution of expelled lithium particles with increasing duration of electron-beam irradiation at IG area and GB area. Dotted lines in **j** are fits.

With increasing electron irradiation time, the accumulation of electrons in/on LLZO creates enough nucleation overpotential to reduce lithium ions to lithium metal (Fig. 4a). During electron-beam irradiation, the secondary electron (SE) or backscattered electron (BSE) signals are collected, allowing the morphology evolution to be recorded. Interestingly, the LLZO surface morphology changes with increasing electron-beam irradiation time (Fig. 4b). After 40 seconds of electron-beam irradiation, three dark lines appeared which meet at one point. We attribute those three dark lines to three grain boundaries. Along these lines, we observed bright features which correspond to metallic lithium expulsions at regular intervals. These expulsions grow with longer electron exposure. After irradiation for around 320 s, there were no additional changes in the morphology of the irradiated LLZO cross-section. At grain boundaries, the expulsions have diameters of (160 ± 10) nm. However, expulsions of much smaller diameters, i.e., (30 ± 3) nm, also appear inside the grains. For comparison, we also irradiated Li_3_PO_4_ amorphous solid electrolytes with electrons. Lithium also expulsed from this material but the distribution was always uniform during nucleation and growth because of the absence of grain boundaries (Supplementary Fig. 19). This proves that grain boundaries play a prominent role in lithium expulsion, nucleation, and growth in polycrystalline inorganic solid electrolytes.

To exclude effects arising from locally inhomogeneous chemical composition, we used an electron beam with the same acceleration voltage and probe current to irradiate samples at a different spot of the cross-section on a larger scale (Fig. 4c). Now, we observed 20 to 30 different grains. The diameter of each LLZO grain ranges from 5 to 10 µm. After electron-beam irradiation, the surface morphology at grain boundaries changed as described above (Fig. 4d).

To determine the composition of the expelled material, we switched from the SE to the BSE detector and imaged the same area again (Fig. 4e, f). In contrast to the SE signal, the BSE signal leads to a dark color at expulsions, whereas the substrate is white. Typically, for a BSE image, a darker color (i.e. a low number of BSE) is caused by lighter elements. Therefore, we attribute the expulsions to lithium, which is the lightest element in LLZO. This conclusion is supported by energy-dispersive X-ray (EDX) spectra of those expulsion particles before and after exposure to air for three minutes. The EDX spectra revealed a higher oxygen content of expulsions after being exposed to air, whereas other elements remained unchanged (Supplementary Fig. 20 and Supplementary Note 5).

A Li_2_CO_3_ contamination layer and surface edges may favor nucleation^12^. However, we performed all our measurements on a freshly prepared and polished LLZO cross-section by strictly eliminating any possible contact with air. Therefore, Li_2_CO_3_ does not cover the LLZO surface (Supplementary Figs. 21 and 22). Surfaces deliberately exposed briefly to air yielded different expulsion results (Supplementary Fig. 23 and Supplementary Note 6). In order to exclude the influence of morphology caused by pellet processing to prepare the fresh sample, we produced a smooth cross-section by argon-ion polishing, which rendered grain boundaries invisible in the SEM. Only voids formed by insufficient fusion between different grains indicated the presence of grain boundaries. With the argon-ion-milled sample, we observed the same phenomenon as for an unpolished sample (Supplementary Fig. 24). Consequently, we attribute the appearance of different expulsion sizes and amounts to different electrochemical properties at LLZO grain boundaries and in-grain areas.

Next, we quantitatively analyzed the size and mass area density of expulsions at grain boundaries and in inner areas of grains. In particular, we observed a contrast in the SEM image near the grain boundaries (Fig. 4g). This area is 300 to 400 nm wide and exhibits almost no extrusions and is larger than LLZO grain boundaries with width below 10 nm^27^. We used this boundary to divide the surface into two kinds of areas: the inner-grain (IG) area with only tiny lithium expulsion particles (average diameter below 20 nm), and the grain boundary (GB) area, including grain boundaries and their adjacent region with no expulsions (Fig. 4h).

We analyzed the size changes of expulsions as a function of time and found that the diameter of expulsions increases with increasing electron dose (Fig. 4i). Interestingly, the first expulsions appeared in the GB area after 40 s. At this electron dose (~8140 e/nm^2^), no features in the IG area could be detected. In general, grain boundaries are usually considered to be favorable sites for heterogeneous nucleation because of energetic reasons^36^. After 360 s, expulsions with diameters of 166 ± 10 nm were observed at grain boundaries. In addition, after 300 s, the increase in diameter slowed down. For the lithium expulsions in the IG area, the average particle radius grew from 25.2 nm at 120 s to 30.2 nm after 400 s. This analysis confirms that grain boundaries act as preferential sites for lithium metal nucleation. More importantly, the grain boundary structure appears to be beneficial for forming larger lithium expulsions.

Next, we compared the mass of expulsion per unit area at the GB area with respect to the IG area (Fig. 4j). We calculated the areal mass density of the lithium expulsions *θ* by

2 $$\theta \left(t\right)=\frac{n\left(t\right)\cdot \frac{4}{3}\pi {r\left(t\right)}^{3}\cdot {\rho }_{{{{{{\rm{Li}}}}}}}}{A},$$

where $n$ is the total number of lithium expulsions, $r$ is the average radius of lithium expulsions, ${\rho }_{{{{{{\rm{Li}}}}}}}$ is the density of lithium metal (0.534 g/cm^3^ at 25 °C) and $A$ is the surface area of the GB or IG. For the lithium expulsions at GB areas, ${\theta }_{{{{{{\rm{GB}}}}}}}$ increases linearly as a function of time from 80 to 320 s. For an electron irradiation time of 320 s, we calculated a ${\theta }_{{{{{{\rm{GB}}}}}},320{{{{{\rm{s}}}}}}}$ of 13.53 × 10^−15^ µg nm^−2^. For the IG area, we observed few expulsions for a duration of 40 s, meaning that *θ* is almost 0. For durations >40 s, ${\theta }_{{{{{{\rm{IG}}}}}}}$ always stays below ${\theta }_{{{{{{\rm{GB}}}}}}}$. After 240 s, ${\theta }_{{{{{{\rm{IG}}}}}}}$ stays constant at a value of ~3.4 × 10^−15^ µg nm^−2^. To compare GB and IG areas, we calculated a ratio of $\frac{{\theta }_{{{{{{\rm{GB}}}}}}}}{{\theta }_{{{{{{\rm{IG}}}}}}}}$ = 4.7 after electron-beam irradiation for 400 s. In other words, there are more lithium expulsions from the GB area than from the IG area.

Time-of-flight–secondary ion mass spectrometry (ToF-SIMS) results show that the lithium elemental concentration at grain boundaries is the same within the in-grain part (Supplementary Fig. 25 and Supplementary Note 7). Therefore, a higher lithium-ion concentration at grain boundaries cannot be the reason for the ratio $\tfrac{{\theta }_{{{{{{\rm{GB}}}}}}}}{{\theta }_{{{{{{\rm{IG}}}}}}}}$ > 1. Combining the fact that no lithium expulsions were observed in regions adjacent to grain boundaries, we conclude that more lithium expulsions at grain boundaries during electron-beam irradiation result from: (1) lithium ions in regions adjacent to grain boundaries move to the grain boundaries, and (2) lithium ions from the LLZO bulk move to the LLZO surface preferably via grain boundaries. We have already shown above that there is no difference in ionic conductivity between grain boundaries and in-grain parts. Thus, the movement of lithium ions to grain boundaries is enabled by additional electrons owing to irradiation, which create a stronger electric field at grain boundaries.

Next, we quantified the expulsion rate based on a model proposed by Saito et al. for ionic conductors^54^:

3 $$\frac{d\theta }{dt}={r}_{1}\sigma E-{r}_{2},$$

where *σ* is the ionic conductivity, *E* is the strength of the electric field created by injected electrons, ${r}_{1}$ is a constant related to the charge transfer resistance of lithium ions being reduced to metallic lithium and ${r}_{2}$ denotes a lithium metal evaporation coefficient. Our SEM measurements were performed in high vacuum (pressure 10^−6^ Pa). Under this condition, the evaporation coefficient ${r}_{2}$ of lithium metal can be ignored^55^. For the lithium expulsions at GB areas, we calculated a lithium expulsion growth rate ${(\frac{d{{{{{\rm{\theta }}}}}}}{{dt}})}_{{{{{{\rm{GB}}}}}}}$ of 0.0399 µg nm^−2^ s^−1^, which is 2.35 times greater than the lithium expulsion growth rate at in-grain areas ${(\tfrac{d\theta }{{dt}})}_{{{{{{\rm{IG}}}}}}}$ between 100 and 320 s. Therefore, ${r}_{1,{{{{{\rm{GB}}}}}}}{\sigma }_{{{{{{\rm{GB}}}}}}}{E}_{{{{{{\rm{GB}}}}}}}$ becomes 2.35 times greater than ${r}_{1,{{{{{\rm{IG}}}}}}}{\sigma }_{{{{{{\rm{IG}}}}}}}{E}_{{{{{{\rm{IG}}}}}}}$. Furthermore, as ${\sigma }_{{{{{{\rm{GB}}}}}}}={\sigma }_{{{{{{\rm{IG}}}}}}}$, probably the larger ${E}_{{{{{{\rm{GB}}}}}}}$ is the possible factor that leads to a larger lithium expulsion rate in the GB region.

**Fig. 5:**
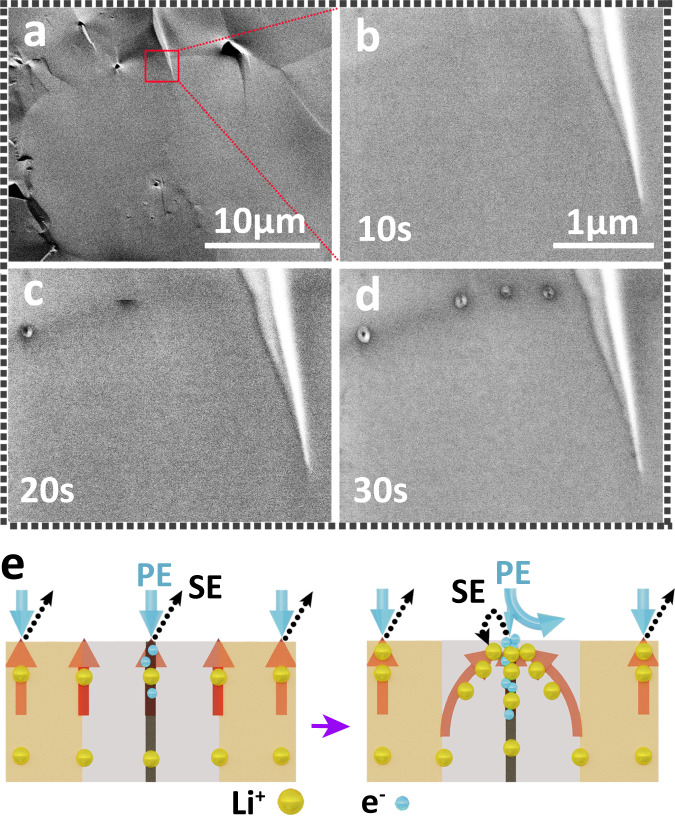
Negative charge effects in grain boundaries. **a** SEM image of a cross-section of LLZO freshly prepared. **b**–**d** Changes of LLZO grain boundaries in the initial stage of electron-beam irradiation. **e** Schematic diagram of the negative charge effect in grain boundaries during electron-beam irradiation.

The presence of a stronger electric field at grain boundaries during electron-beam irradiation is also supported by the contrast at grains that developed during SEM imaging. We observed that, at the initial stage of electron-beam irradiation, there is almost no contrast between GB and IG areas in SE images, see Fig. 5a. After electron-beam irradiation for 10, 20, and 30 s, the contrast develops as shown in Fig. 5b–d, respectively. The grain boundaries gradually become darker than the in-grain parts. This means fewer secondary electrons are emitted from grain boundaries than from in-grain parts. We exclude an initially increased number of positive charges, i.e., lithium ions, at the GB area as there is no difference in ionic conductivity between grain boundaries and in-grain parts. Thus, a locally higher electron concentration at grain boundaries prevents the generation of SE by deflecting primary electrons (PE)^56^. Electrons accumulate at the grain boundaries, which attract positive lithium ions from adjacent regions that move to grain boundaries. Also, those lithium ions attract a certain percentage of secondary electrons (Fig. 5e). This particular phenomenon is not related to surface roughness (Supplementary Figs. 26 and 27). Changes, in contrast, could arise from irreversible reactions or changes in surface characteristics. We exclude this possibility by switching the electron beam off for 30 min. The dark contrast at grain boundaries gradually disappeared in the same area. Therefore, the dark contrast at grain boundaries results from electron accumulation (Supplementary Fig. 28 and Supplementary Note 8).

### Model for the electron-beam irradiation experiment

**Fig. 6:**
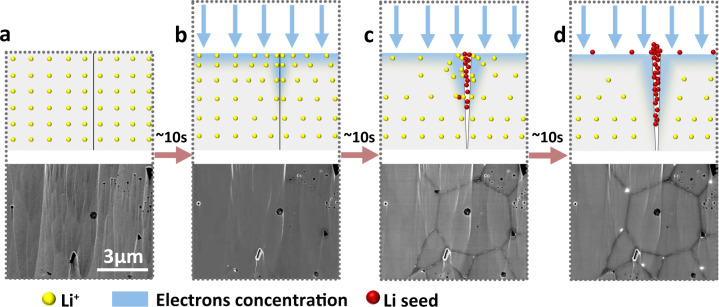
Lithium expulsions formation process at grain boundaries. **a**–**d** Schematic diagram of the formation of metal-lithium particles on LLZO surface during electron-beam injection with a time step around 10 s.

Using the findings presented above, we propose a model to explain how an electric field causes extended formation of lithium metal at grain boundaries on LLZO surface (Fig. 6a). The analysis of the electron-beam irradiation experiments reveals that electrons preferentially cause lithium metal nucleation and growth at grain boundaries. The higher electron concentration at grain boundaries leads to a higher electron concentration gradient from grain boundaries to their adjacent regions, which creates an electric field that attracts adjacent lithium ions (Fig. 6b). The electric field increases with ongoing electron irradiation. Upon reaching a certain negative potential, lithium ions move towards the grain boundaries, where they are reduced to lithium nuclei as denoted by the red particles in Fig. 6c. With an increasing amount of reduced lithium, mechanical strain builds up at grain boundaries. Such mechanical strain extrudes lithium from the surface of LLZO (Fig. 6d). In the IG area, the lithium expulsion rate is lower, and the diameters of the final expulsions are smaller and uniformly distributed. In this case, electrons are homogeneously distributed at the LLZO bulk surface.

### Model for lithium-dendrite growth along grain boundaries

We have analyzed the electrical behavior of LLZO samples by *operando* KPFM, tr-EFM, and SEM to understand the dynamics of preferential lithium nucleation and expulsion in and from grain boundaries. Local tr-EFM and KPFM measurements indicate that grain boundaries do not provide a preferential channel for lithium ion transport. Thus, we suggest that lithium-ion transport is homogeneous inside LLZO sintered in an oxygen atmosphere (under a 150-sccm oxygen flow) without any pressure being applied on. However, our measurements and analyses also indicate that electron transport in LLZO is non-homogeneous near the lithium electrode. We conclude from our operando KPFM and electron-beam irradiation results that the grain boundaries near the (plating) lithium electrode become more resistive for ions. The latter results from accumulation of electrons at grain boundaries causing a space charge layer-like structure. This cannot be observed in amorphous solid electrolyte Li_3_PO_4_ without grain boundaries.

We elucidate that grain boundaries trap excess electrons from the plating electrode. DFT simulations suggest a smaller band gap in LLZO grain boundaries relative to the bulk^18^, which could be the origin of trapping excess electrons (in chemical terms: trapping excess Li^0^). Those excess electrons could cause space charge layer formation at grain boundaries causing a step-like CPD signal.

**Fig. 7:**
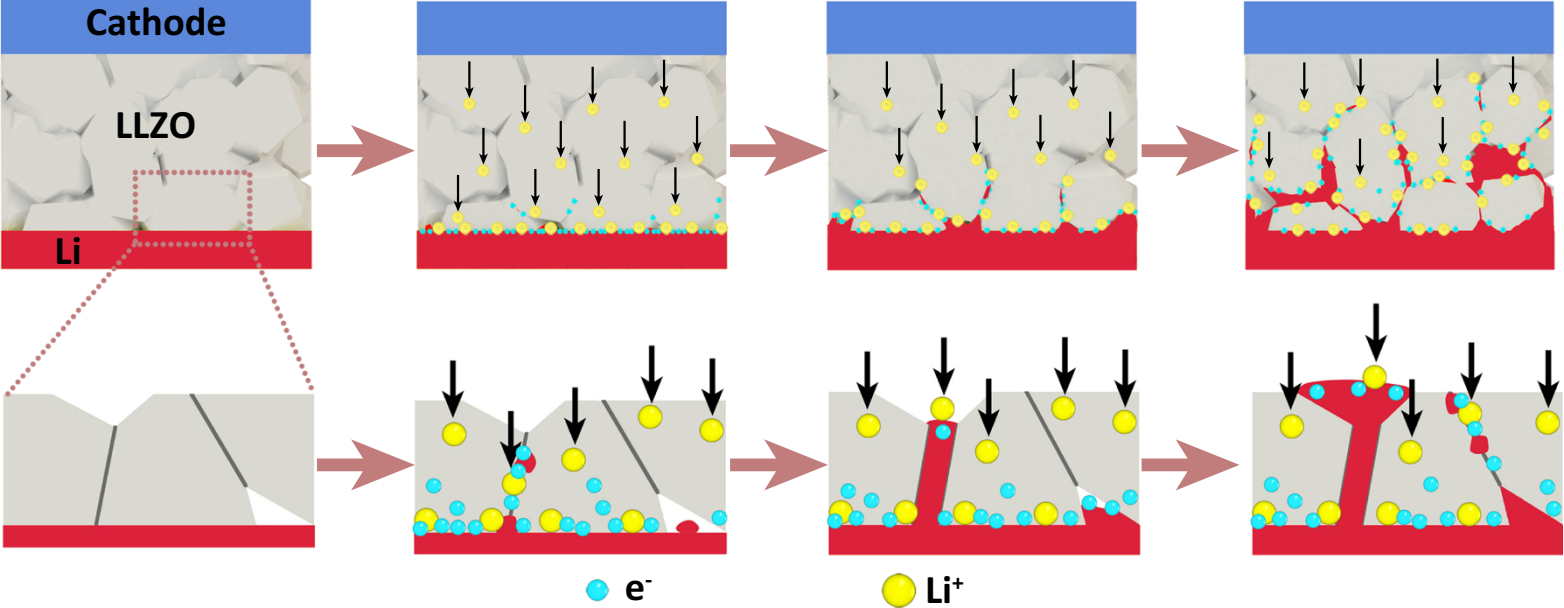
Proposed mechanism for Li dendrite nucleation and growth in all-solid-state asymmetric Li metal cells. With time increasing, lithium dendrites continuously nucleate at and growth along grain boundaries from anode side to cathode side.

As a consequence of this space charge layer, lithium metal penetrates into LLZO. In fact, dendrites are often observed primarily at grain boundaries after cycling all-solid-state symmetric cells with an LLZO solid electrolyte separator (Supplementary Fig. 29). Based on these assumptions, we speculate that during the charging process of a Li metal battery containing an LLZO electrolyte and a high-voltage positive electrode (cathode), lithium ions are extracted from the cathode and move to the lithium anode (Fig. 7). At the same time, the lithium negative electrode (anode) provides electrons to the LLZO solid electrolyte surface, thus reducing lithium ions to lithium at the Li|LLZO interface. Near the anode Li|LLZO interface, lithium metal grows through voids and defects in the LLZO. When these primary lithium dendrites reach grain boundaries, they inject electrons preferentially to the grain boundaries owing to their higher acceptance of electrons. With progressing duration or increasing current density, the electron concentration locally increases within grain boundaries (Supplementary Fig. 10 and Supplementary Fig. 30). Thus, grain boundaries near the plating lithium electrode areas (in symmetric Li|LLZO|Li cells) reach the overpotential for lithium-ion reduction earlier than other parts in the entire LLZO. Then formed lithium dendrites continue to grow along other grain boundaries, again due to the high grain boundary electron trapping ability, leading to easier lithium ion reduction. Because of the limitation of KPFM scanning area and the randomness and uncertainty in lithium dendrite growth in LLZO, we can only observe the initial grain boundary effect on electron transport close to the plating Li-CE but not track the whole lithium growth process. In addition, the growth can be enhanced by a high stack pressure applied on the solid electrolyte. The high stack pressure may locally exceed the yield stress in the material which then leads to additional voids and cracks formation^57^. In those voids and cracks, lithium can further nucleate and grow, and dendritic growth continues. The lithium dendrites gradually fill voids, defects, and grain boundaries. Finally, this process continues along grain boundaries until dendrites reach the cathode. As a result, the battery short-circuits.

This growth mode is similar to the one derived by phase field simulation results based on DFT calculation performed by Tian et al^18^. Cubic-Li_7_La_3_Zr_2_O_12_ grain surfaces and grain boundaries are prone to trap excess surface electrons. The phase field simulation shows that lithium dendrites first form at grain boundaries close to Li-CE – as our KPFM measurements also suggest. With increasing lithium plating time, lithium dendrites grow faster along grain boundaries because the high electron concentration at grain boundaries promotes lithium ion reduction^55,58,59^.

Our model explains important aspects of the dynamic and preferential growth of lithium dendrites at grain boundaries. In particular, our operando measurements and analysis indicates that growth of lithium dendrites starts at LLZO grain boundaries near the lithium anode, and that the electronic conduction properties of grain boundaries play a major role in this process. Understanding lithium dendrite (or filamentary) growth is important to prevent failure of lithium metal all-solid-state batteries. Our findings indicate that the interface to the lithium anode is of particular importance for suppressing dendritic growth of lithium. Adding a functional layer at this interface there could be the key to suppressing electron uptake along grain boundaries, thereby preventing dendritic growth of lithium.

## Methods

### LLZO preparation and characterization

To synthesize garnet-type solid electrolyte Li_6.25_Al_0.25_La_3_Zr_2_O_12_ (LLZO) Li_2_CO_3_ (>99.0%, Sigma-Aldrich), La(OH)_3_ (99.9%, Sigma-Aldrich), Al_2_O_3_ (99.8%, abcr), and ZrO_2_ nano-powder (<100 nm, Sigma-Aldrich) were mixed in a stoichiometric ratio. This mixture was then ball-milled for 12 h at 350 rpm under pure oxygen atmosphere. After milling, the homogenized powder was pressed into large pellets in MgO-crucibles and then calcined at 1000 °C for 4 h under a 150-sccm oxygen flow. After ball-milling again for 20 h at 350 rpm, small pellets with diameters of ~8.3 mm and thicknesses of 2 mm were isostatically pressed with 380 MPa and sintered at 1230 °C in MgO-crucibles with mother powder (calcined LLZO powder) under oxygen flow. The LLZO pellets have a porosity of ~(94 ± 2)%, their ionic conductivity is (4.6 ± 0.4)10^−4^ S cm^−1^. The ionic conductivity is calculated by considering the thickness, the contact area with the electrode, and the total bulk and grain boundaries resistance of the LLZO pellet in a Li|LLZO|Li symmetric cell extracted by fitting of electrochemical impedance result which is acquired in at 25 °C (supplementary Fig. 2). The sintered LLZO was characterized with x-ray diffraction by using a PANalytical Empyrean powder diffractometer in Bragg-Brentano θ-θ geometry with copper K_α_ radiation.

#### Cell fabrication

1) Both Li|LLZO|Li symmetric and Li|LLZO|Au Hebb-Wagner cells were prepared in a glove box filled with argon gas (purity 99.9999%, *p*(H_2_O)/*p* < 0.1 ppm and *p*(O_2_)/*p* < 0.1 ppm). The schematic structure of both kinds of cells is shown in Supplementary Fig. 31.

For a Li|LLZO|Li symmetric cell, first, the surface of LLZO with a diameter of 8.35 mm was polished with 1200-grit SiC sandpaper (Buehler, CarbiMet) to smooth the surface and remove the surface passivation layer. Then, the lithium electrodes were fabricated by pressing freshly prepared lithium metal sheets at an isostatic pressure of 400 MPa in an oil-press protected by three sealed polymer layers onto the two opposite, polished faces of the LLZO pellet. The used lithium sheets were prepared prior by removing the native passivation layer of a lithium metal chunk (100% lithium, TMAX) with a ceramic knife and subsequent pressing to a thin foil. The diameter and thickness of the lithium metal electrode were approximately 6 mm and 0.1 mm, respectively.

For the Li|LLZO|Au Hebb-Wagner cells, the LLZO surfaces were polished as well. Then, on one side a lithium electrode was pressed as described above. On the opposite side, a Au electrode was sputtered under vacuum (<2 × 10^–5^ Pa) (DC magnetron sputtering device, Bal-Tec MED 020) with a DC current of 40 mA. The thickness of the Au layer was around 100 nm.

2) The Li|Li_3_PO_4_|Li symmetric cell was fabricated in a homemade vacuum deposition system. A 2 µm thick Li layer was first deposited on an Al_2_O_3_ substrate by thermal evaporation (Ar environment at a pressure of 0.74 Pa). Then, the Li_3_PO_4_ solid electrolyte (2.5 µm) was deposited by RF magnetron sputtering (Ar environment at a pressure of 0.15 Pa and DC power of 100 W). Finally, the other Li metal anode (2 µm) was deposited on the Li_3_PO_4_ by thermal evaporation.

#### Electrochemical measurements

Electrochemical performance measurements were carried out in an Ar glove box (*p*(H_2_O)/*p* < 0.1 ppm and *p*(O_2_)/*p* < 0.1 ppm) with a potentiostat (SP150, Biologic) under room temperature 23 ± 2 °C. We applied an external current to one lithium electrode as the working electrode (WE). The other lithium electrode was connected to ground and acted as the counter electrode (CE). We did not apply external pressure on the cell during electrochemical measurements.

1) Electrochemical impedence spectroscopy (EIS): EIS was carried out within the frequency range of 3 MHz to 0.1 Hz with an amplitude of 10 mV and 6 data points per frequency decade. Fitting was performed using the software RelaxIS 3. We used an equivalent circuit of two parallel *R*_i_*-C*_i_-elements in series, where *R*_i_ and *C*_i_ denote resistance and capacitance of the bulk and grain boundary transport processes. A 30 second OCV measurement was carried out prior to measuring to secure that the system is in a relaxed state.

2) Constant current/potential measurements: For the Li|LLZO|Li and Li|Li_3_PO_4_|Li symmetric cells, the Li-WE was connected to the WE of the potentiostat. The Li-CE was connected to the CE of the potentiostat and connected to ground. Then, different currents or potentials were applied on Li-WE to drive lithium ions from Li-WE to Li-CE. At the same time, operando KPFM is carried on the surface of LLZO and Li_3_PO_4_.

For the Li|LLZO|Au Hebb-Wagner cell, the Au electrode was connected to the WE of the potentiostat. The Li electrode was connected to the CE of the potentiostat (thus connected to ground). Then, different potentials were applied to the Au electrode to polarize the Hebb-Wagner cell. At the same time, operando KPFM was carried out on the LLZO surface. After that, the electrical connection was switched. The Li electrode is connected to the WE and the Au CE is connected to ground. Then again KPFM measurements were performed.

#### Argon-ion milling

For SEM and SFM experiments, we prepared smooth cross sections by argon-ion milling (IM4000, Hitachi). First, we broke the cell into two pieces along the diameter of the sample. Then, half of the broken cell was fixed in a cross-section milling holder. Next, a 100-µm-thick stainless-steel mask was placed in front of the lithium electrode to define the position for argon-ion milling (Supplementary Figs. 3 and 32). Ion milling was performed at an acceleration voltage of 2 kV with a discharge current of ~450 µA. During argon-ion milling, the sample stage rotated at a speed of 30 rpm. The polishing process took ~14 h. After ion milling, we used the air protection system of the argon-ion milling machine to transfer samples from the ion-milling chamber to the argon glove box. Typically, the surface root mean square (RMS) roughness after argon-ion milling was ~20 nm at an area of 5 × 5 µm^2^ measured by means of SFM.

#### Scanning force microscopy

All scanning force microscopy (SFM) measurements including operando KFPM and tr-EFM (MFP-3D Asylum Research, Oxford Instrument, USA)) were performed in a glove box (GS, Germany) filled with argon (purity 99.9999%). The inert argon gas environment prevents battery degradation, including LLZO and lithium electrodes reacting with O_2_, N_2_, CO_2_, N_2_, and H_2_O. SFM measurements were taken with PtIr-coated cantilevers, having a nominal spring constant of 2 N/m and a nominal resonance frequency of 75 kHz (SCM-PIT-V2, Bruker, USA). A homemade holder was used to fix and connect the battery sample to the potentiostat. The potentiostat was operated outside the glove box with electrical connections through the glove box wall. Photographs of the equipment are shown in Supplementary Fig. 33.

1) Operando KPFM: KPFM measurements were carried out in heterodyne frequency modulation (FM) mode with an external lock-in amplifier (Zurich Instruments HF2LI-MOD) to measure contact potential difference (CPD) between SFM tip and sample. More details about the KPFM working principle and interpretation is provided in Supplementary Note 9.

We first performed KPFM measurements on the LLZO or the Li_3_PO_4_ surface at position *x* without any external potential applied to the Li-CE. *x* is the distance to the Li-CE. Upon applying a constant external current or potential to the symmetric Li|LLZO|Li cell, we measured a CPD change at a specific position *x* on LLZO surface. It is defined as:

4 $${{CPD}\left(x\right)}_{{{{{{\rm{applied}}}}}}}-{{CPD}\left(x\right)}_{{{{{{\rm{OCV}}}}}}}=k\left(x\right)\cdot {\phi }_{{{{{{\rm{applied}}}}}}}+\triangle \Phi \left(x\right),$$

Here, ${{CPD}\left(x\right)}_{{{{{{\rm{OCV}}}}}}}$ is the contact potential difference in the OCV state. ${{CPD}\left(x\right)}_{{{{{{\rm{applied}}}}}}}$ is the contact potential difference with an applied potential ${\phi }_{{{{{{\rm{applied}}}}}}}$. $\triangle \Phi \left(x\right)$ is work function change of the LLZO surface at position *x*, which changes with a material’s composition change.

2) Tr-EFM: Tr-EFM measurements were performed with the external lock-in amplifier (Zurich Instruments HF2LI-MOD) as well. Details are provided in Supplementary Note 10. Briefly, we performed tr-EFM measurements as follows. First, the cantilever was excited to vibrate at its first resonance frequency with an internal piezoelectric oscillator. A feedback electronic regulates the height between tip and measured points on the sample by keeping the vibration amplitude at the first resonance frequency constant. In this way, a topographic image of the sample was obtained in tapping mode. In addition, we applied an AC voltage at the second resonance frequency to generate an electrostatic force between SFM tip and the LLZO sample.

Next, we brought the tip into contact with the surface and applied a DC bias voltage of −3 V for 1.5 s to induce ion displacements in LLZO. Then, we grounded the tip for 1.5 s to allow ion relaxation, after which we retracted the tip and repeated this sequence at the next position. During this sequence, we recorded the vibration amplitude of the cantilever at its second resonance frequency (*ω*) to track changes of the electrostatic force ${F}_{{{{{{\rm{es}}}}}}({{{{{\rm{t}}}}}},{{{{{\rm{\omega }}}}}})}$. As lithium ions are the main mobile charges in LLZO, we attribute changes of the electrostatic force to lithium-ion displacement. In solid ionic conductors, the ionic transport follows a stretched-exponential time response due to the electric field between the sample and tip^60–63^:

5 $$\Delta {A}_{({{{{{\rm{t}}}}}},\omega )}=\Delta {A}_{{{{{{\rm{slow}}}}}}}{{{{{\rm{exp }}}}}}\left[-{({{{{{\rm{t}}}}}}/\tau )}^{\beta }\right]+\Delta {A}_{{{{{{\rm{fast}}}}}}},$$

Here, $\Delta {A}_{({{{{{\rm{t}}}}}},\omega )}$ is the total amplitude change at frequency *ω*, $\Delta {A}_{{{{{{\rm{fast}}}}}}}$ is the amplitude change, before ionic relaxation due to ultrafast vibrational and electronic polarization^44,64^, $\Delta {A}_{{{{{{\rm{slow}}}}}}}$ is the amplitude change until the system reaches a saturation state due to ionic relaxation, *τ* is a time constant and *β* is a stretch exponent representing ion diffusion properties^44^. For simplicity, we set *β* to 1 to fit the $\Delta {A}_{({{{{{\rm{t}}}}}},\omega )}$ vs. time curve. Differences in ion diffusivity ($D \sim 1/\tau$) can be measured by fitting Eq. (5) to measurements recorded at different positions on a freshly prepared LLZO surface^65^.

The comparison of tr-EFM results on LLZO and Au shows that tr-EFM can effectively track ion diffusivity in ionic conductors (Supplementary Fig. 34). The magnitude of the applied DC voltage on the tip does not alter the calculated relaxation time of the sample (Supplementary Fig. 35).

#### Electron beam irradiation and scanning electron microscopy (SEM)

Electron beam irradiation experiments and SEM measurements were performed in a field-emission SEM (Hitachi SU8000). To avoid surface contamination or reaction with air of LLZO, a transport sealing box filled with argon gas (purity 99.9999%) was used to transfer samples from the glove box into the instrument. The LLZO pellet was fixed to an SEM sample holder, which allowed the investigation of the cross-section in vertical direction. A region on the LLZO surface was selected by SEM standard imaging mode using the secondary electron detector which shall be irradiated with electrons. Then, this region was irradiated with the electron beam using an acceleration voltage of 5 kV, leading to a probe current of ~1 nA. At the same time, we followed changes in the surface by reading out the secondary electron detector or the BSE detector.

#### Time-of-flight–secondary ion mass spectrometry (ToF-SIMS)

We used ToF**-**SIMS (IONTOF TOF.SIMS NCS) to characterize the lithium element and related molecular information on the surface of LLZO, especially lithium and its related molecular species. Bi^3+^ ions with an energy of 30 keV served as the primary ion source. To reduce surface roughness, the LLZO cross-section was first polished by argon-ion milling as described above. Then we used a transfer chamber filled with pure argon gas to protect the LLZO cross-section during the transfer into the ToF**-**SIMS chamber. The LLZO sample was installed in the ToF**-**SIMS chamber and then the chamber was evacuated to high vacuum (<10^−5^ mbar). The LLZO was exposed to air for less than one minute while the sample was being installed in the ToF**-**SIMS chamber.

### Reporting summary

Further information on research design is available in the Nature Portfolio Reporting Summary linked to this article.

## Supplementary information

Supplementary Information

Reporting Summary

## Data Availability

The data for all measurements used in this study are available in the Edmond database of the Max Planck Society at 10.17617/3.GOCS2D.
