# Supplementary material for: Understanding the evolution of lithium dendrites at Li6.25Al0.25La3Zr2O12 grain boundaries via operando microscopy techniques
Source: Nat Commun. 2023 Mar 9;14:1300. doi: 10.1038/s41467-023-36792-7 (PMC9998873; doi:10.1038/s41467-023-36792-7)
Supplement: Supplementary file 1 — Supplementary Information [file 41467_2023_36792_MOESM1_ESM.pdf]

SUPPLEMENTARY INFORMATION

Understanding the evolution of lithium dendrites at the $Li_{6.25}Al_{0.25}La_3Zr_2O_{12}$ grain boundaries via operando microscopy techniques

Chao Zhu[1], Till Fuchs[2], Stefan A.L. Weber[1,3], Felix. H. Richter[2], Gunnar Glasser[1], Franjo Weber[1], Hans-Jürgen Butt[1], Jürgen Janek[2] and Rüdiger Berger[1*]*

1. *Max Planck Institute for Polymer Research, Ackermannweg 10, 55128 Mainz, German*
2. *Institute of Physical Chemistry & Center for Materials Research, Justus Liebig University Giessen, Heinrich-Buff Ring 17, 35392 Giessen, German*
3. *Institute of Physics, Johannes Gutenberg University Mainz, Staudingerweg 7, 55128 Mainz*

Email address: Juergen.Janek@phys.Chemie.uni-giessen.de; berger@mpip-mainz.mpg.de

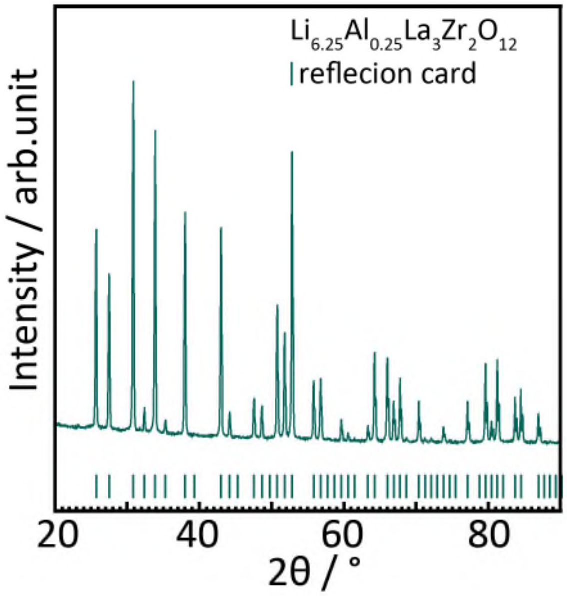

Supplementary Figure 1. LLZO structural characterization. X-ray diffractogram of the herein prepared LLZO pellet displayed together with a reflection card of said phase.[1] No impurities or other crystalline phases are observed.

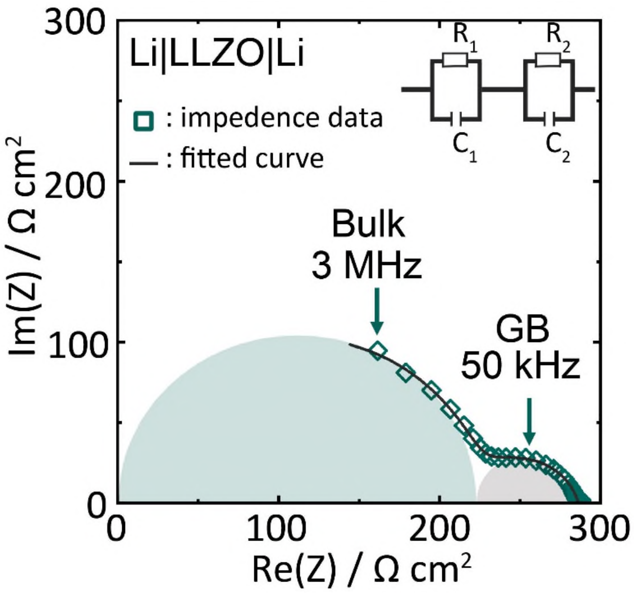

Supplementary Figure 2. Electrochemical impedance spectroscopy characterization of the Li|LLZO|Li symmetric cell. Nyquist-Plot with data depicted as symbols and a corresponding fit as a line of a herein prepared Li|LLZO|Li cell showing only bulk and grain boundary contributions to the transport, as the interfacial resistance is negligible. The impedance result was acquired at room temperature under open circuit voltage of the cell, which was fitted using a serial connection of two parallel resistance (R) – constant phase element (C) circuit elements representing bulk and grain boundary transport. Supplementary Table 1 provides the respective fitted values and errors.

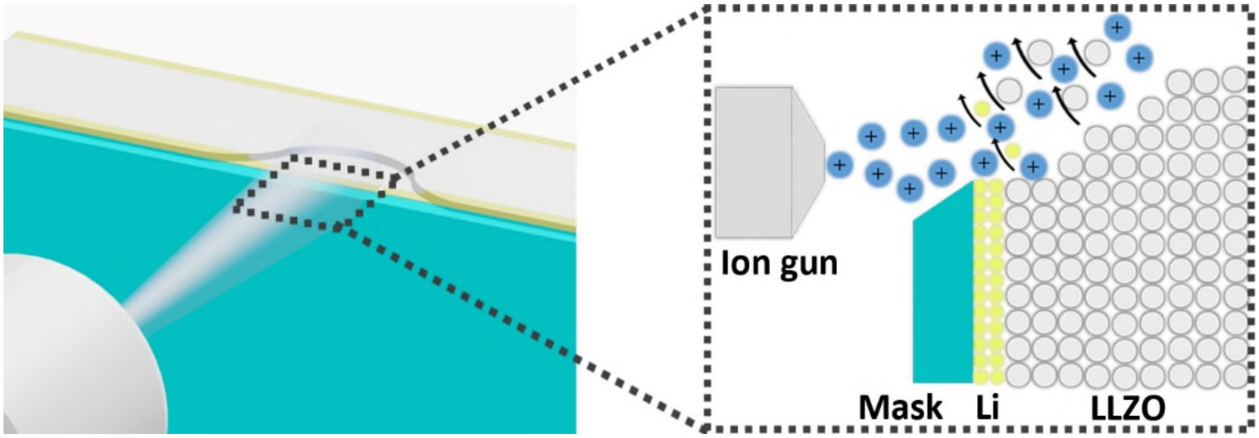

Supplementary Figure 3. Argon ion milling schematic diagram. Schematic 3D representation of the argon ion milling direction and its atomistic process for the Li|LLZO|Li cell in side view.

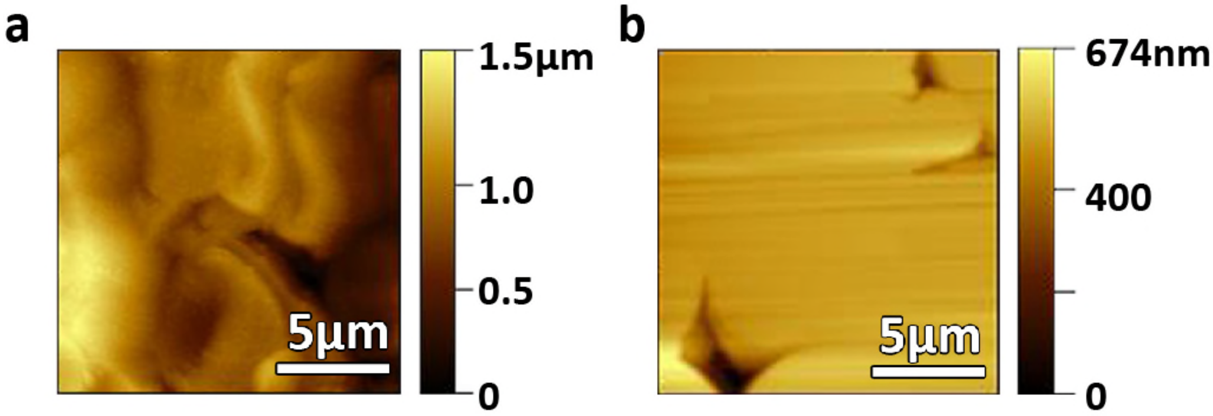

Supplementary Figure 4. Topography comparison of a broken and a polished LLZO surface. Topography measured by SFM. **a** Cross section of LLZO that was obtained by only breaking the cell into two pieces. This procedure results in a root mean square surface roughness of 354 nm. **b** Cross section of LLZO that was obtained by breaking the cell into two pieces and by a subsequent polishing step done by Argon-ion milling. This subsequent polishing step reduces the root mean square surface roughness to 54 nm.

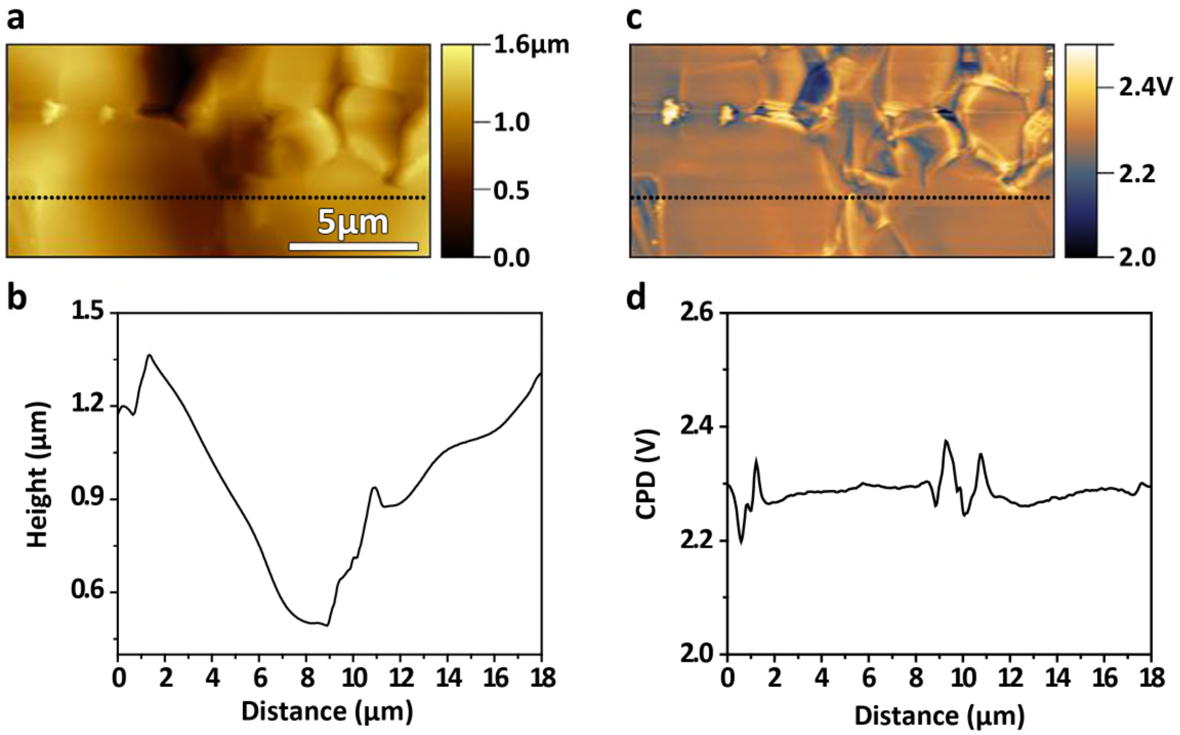

Supplementary Figure 5. KPFM measurement of a freshly broken LLZO surface. We obtained the LLZO sample by breaking the cell into two pieces in argon filled glovebox. Directly afterwards we did KPFM measurements on the cross section in the same argon filled glovebox. The KPFM measurement shown was taken 5 min after breaking the cell. **a** Topography of the LLZO cross section. **b** Topography line profile along the dotted line indicated in **a**. **c** Simultaneous obtained CPD map of the same area as shown in **a**. **d** CPD line profile along the dotted line indicated in **c.**

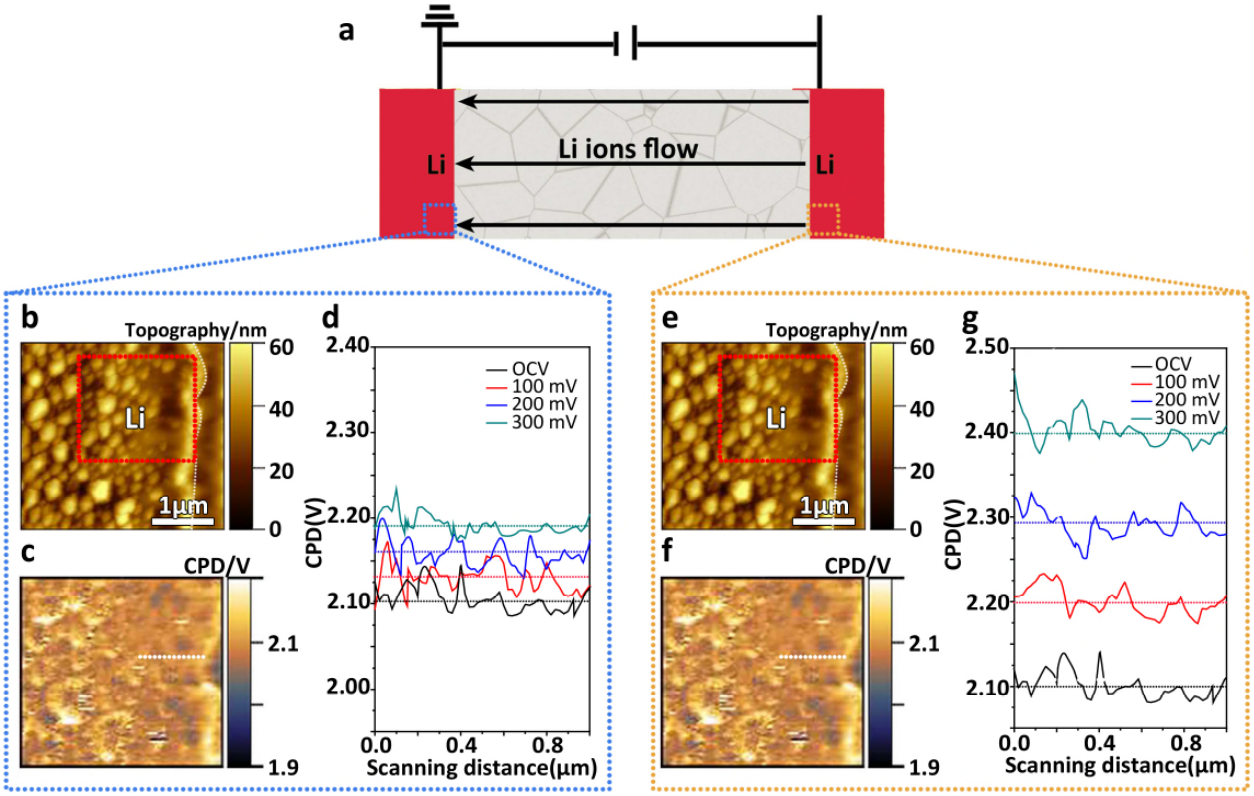

Supplementary Figure 6. KPFM measurement on the lithium electrode close to Li|LLZO interface. a Diagram of the Li|LLZO|Li symmetrical cell. **b** Topography of the lithium electrode surface used as CE, which is outlined with a blue dashed square in **a**. **c** The CPD map of the area within the red dashed box in **b** under OCV state. **d** Line profiles of the CPD along the white dashed line in **c** recorded under different applied potentials between the Li-WE and the Li-CE electrodes. **e** Topography of the lithium electrode surface used as WE. In order to make the results comparable, we measured at the sample place but changed from the Li-WE to the Li-CE by reversing the electrical connection. Thus, the CPD map at OCV shown in **c** and **f** are similar. **g** Line profiles of the CPD along the white dashed line in **f** recorded under different applied potentials between the Li-WE and the Li-CE electrodes.

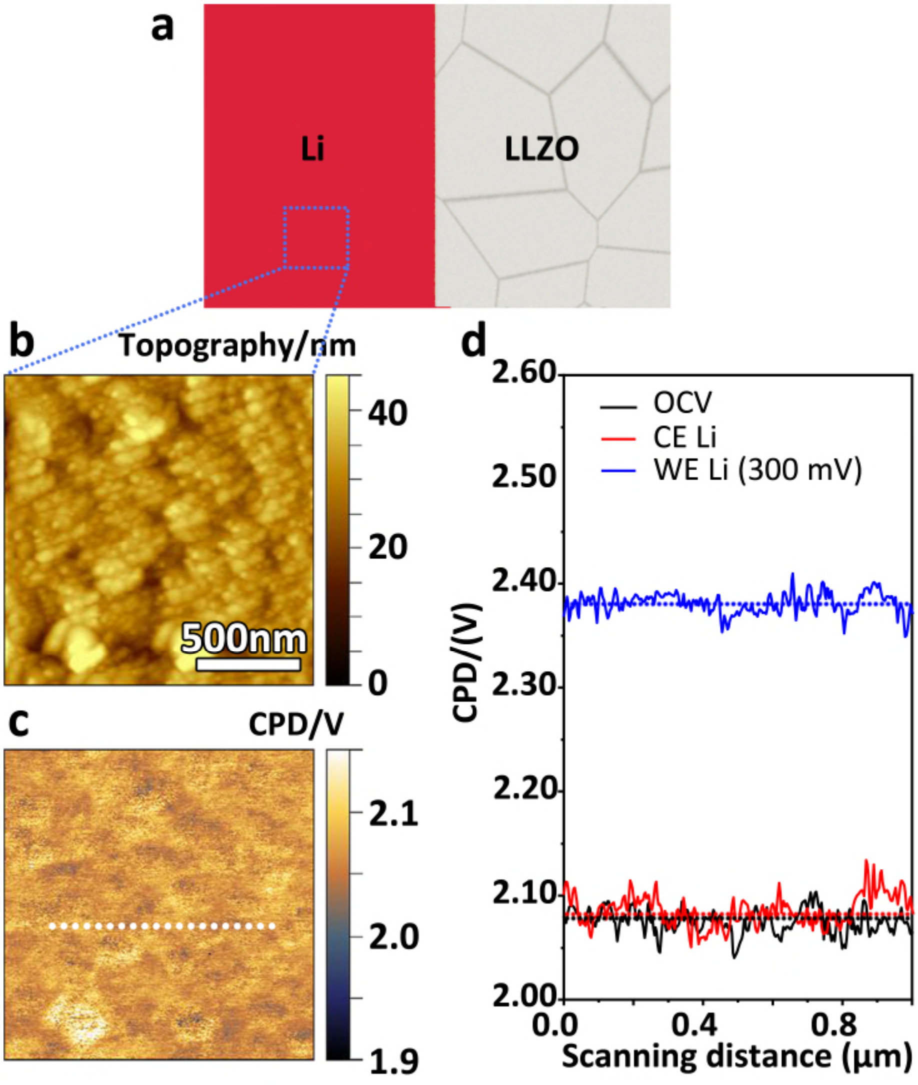

Supplementary Figure 7. KPFM measurement on the lithium electrode far from the Li|LLZO interface. a Diagram of the lithium electrode and LLZO solid electrolyte. **b** Topography of the area which is indicated by the dashed square in **a**. **c** The CPD of the corresponding area in **b**. **d** The measured CPD line profile along the white dashed line in **c** under applied potential of 300 mV between the Li-CE and the Li-WE electrodes.

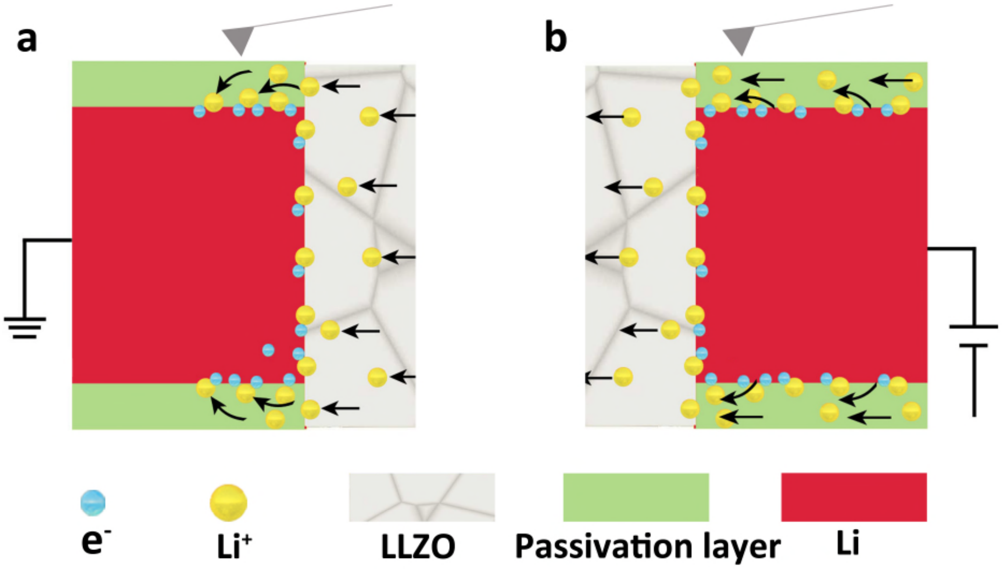

Supplementary Figure 8. Schematic representation of the CPD increase of the lithium electrode close to the Li|LLZO interface. On the lithium electrodes (red color) a thin passivation layer might be present (green color). The passivation layer can transport ions as depicted in the schematic picture. **a** and **b** shows Li-ion transport at the Li-CE and Li-WE, respectively.

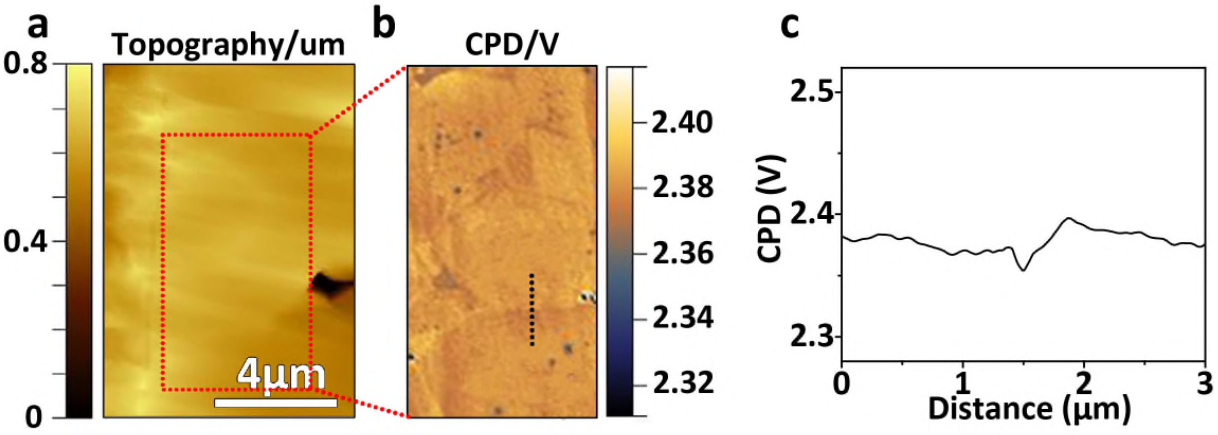

Supplementary Figure 9. Additional KPFM measurement of LLZO at the same position as shown in Figure 1. a Topography of LLZO at the same position as shown in Figure 1 (Li-CE|LLZO interface region). **b** The corresponding CPD map of the area outlined in the red dotted box. **c** Averaged line profile over a width of 100 nm extracted along the black dotted line shown in **b**.

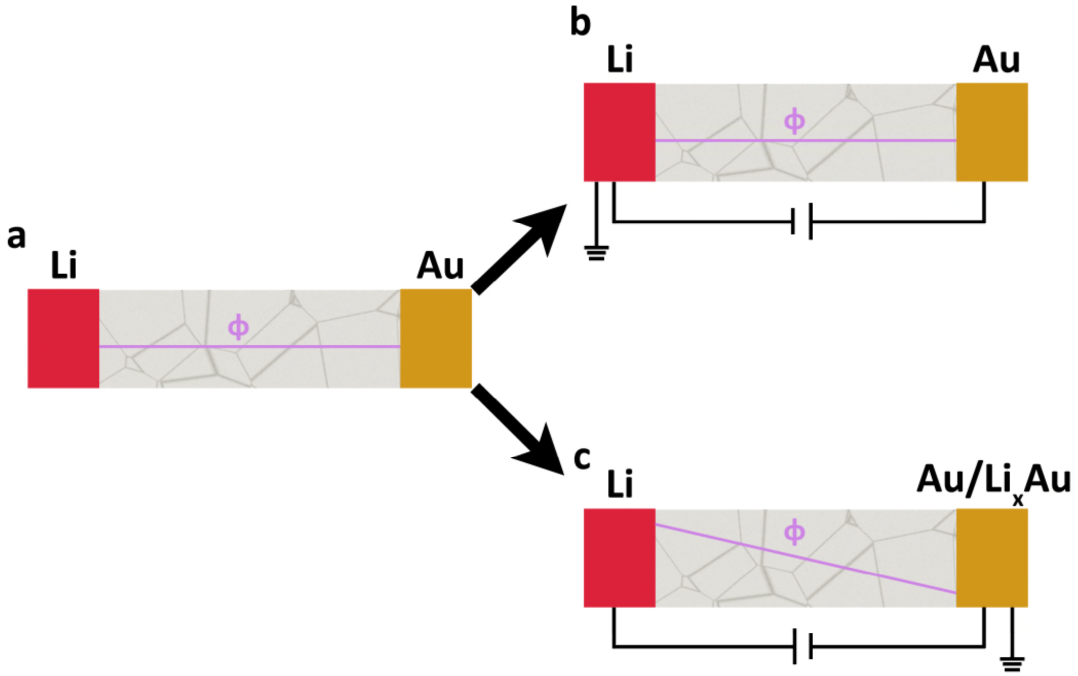

Supplementary Figure 10. Inner (Galvani) potential changes of LLZO in a Hebb-Wagner cell. Schematic profile of the Galvani potential ϕ (pink dotted line) in Hebb-Wagner cell (Li|LLZO|Au). **a** In a pristine state. **b** In a state where a positive DC voltage is applied to the gold electrode and the cell is polarized. **c** In a state where a positive DC voltage is applied to the lithium electrode.

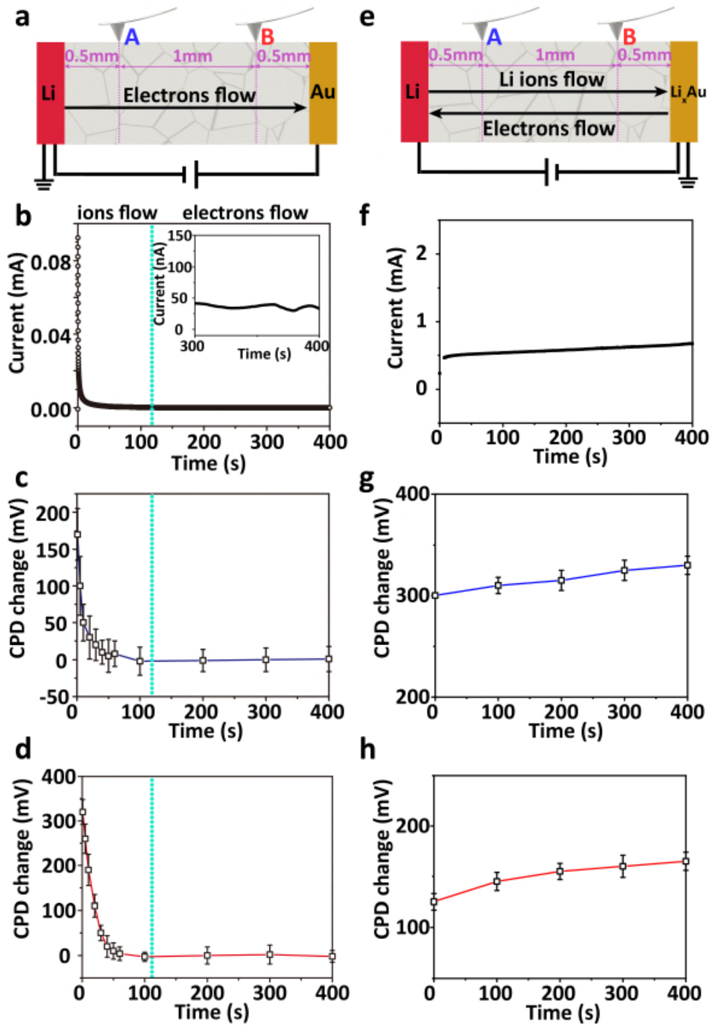

Supplementary Figure 11. KPFM measurements on the LLZO surface in a Hebb-Wagner cell. We performed two different polarization experiments with a Hebb-Wagner cell composed of Li|LLZO|Au. **a** The gold electrode is connected to a positive DC voltage and constitutes an ion blocking operation, while electron can still be transported. **b** displays the corresponding current vs. time dependence at a DC voltage of 500 mV. The inset in **b** shows the current arising from electrons, which is on a scale of 20 nA. **c** displays the corresponding CPD changes with time at point A on the LLZO surface for electrical connection at a DC voltage of 500 mV. **d** corresponds to the CPD changes at point B on the LLZO surface. The blue dashed line in **b**, **c** and **d** divides the time region to a ion flow domain and a electron flow domain. The CPD signals in **c** and **d** approach towards zero in electrons flow domain region. Thus no Galvani potential difference exists along the LLZO. **e** The lithium electrode is connected to a positive DC voltage. Now lithium ions flow to the Au electrode and electrons flow to the lithium electrode, i.e. non-blocking. **f** displays the corresponding current vs. time dependence at a DC voltage of 500 mV. The current is much higher due to the lithium ion current compared to the ion blocking condition **b**. **g** displays the corresponding CPD changes with time at point A on the LLZO surface at a DC voltage of 500 mV. **h** corresponds to CPD changes at point B on LLZO surface. The error bars in **c**, **d**, **g** and **h** are calculated by three independent experiments.

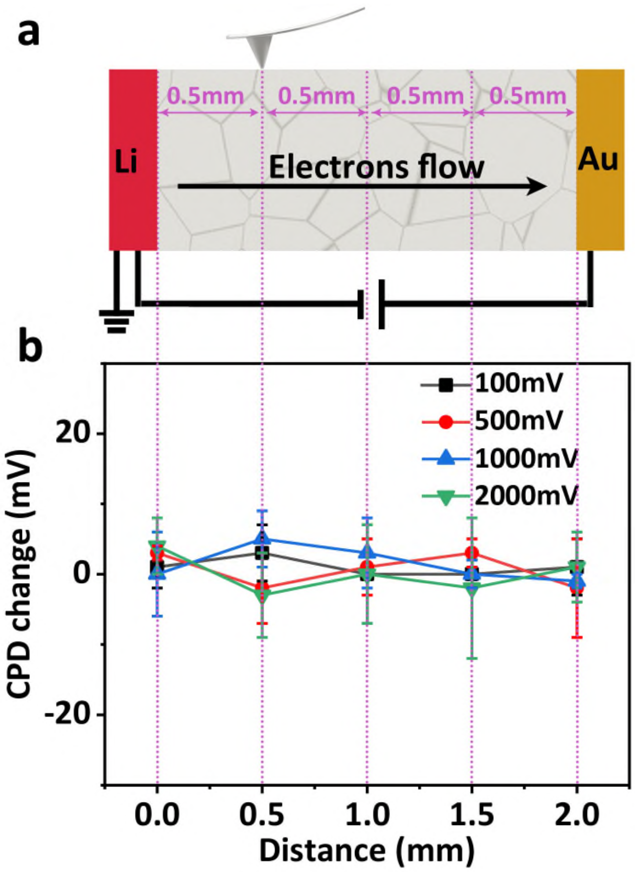

Supplementary Figure 12. KPFM measurement on a LLZO surface in a Hebb-Wagner cell. a We applied different DC voltages to the gold electrode of a Hebb-Wagner cell (Li|LLZO|Au). **b** CPD changes measured on LLZO at different distances from the lithium electrode at different DC voltages. The CPD changes is taken after stable polarization states were reached, respectively. The error bars corresponds to three independent experiments.

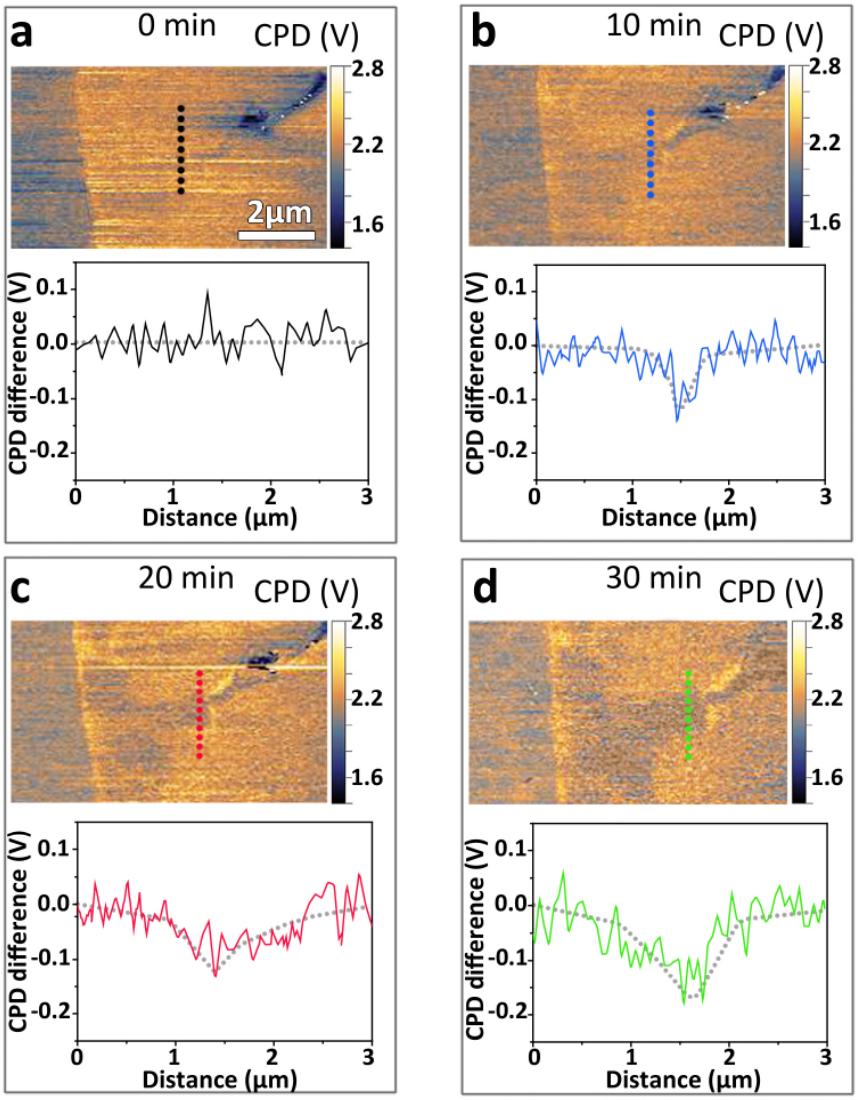

Supplementary Figure 13. CPD evolution across a LLZO grain boundary with increasing time under polarization. Time dependent CPD of the Li-CE|LLZO interface region while applying a 300 mV potential between the Li-CE and Li-WE. CPD measurement at **a** 0 minutes, **b** 10 minutes, **c** 20 minutes and **d** 30 minutes. Below each CPD map, we plotted the CPD difference along the dotted lines, respectively. The CPD difference was calculated by subtracting the CPD value measured at the OCV state.

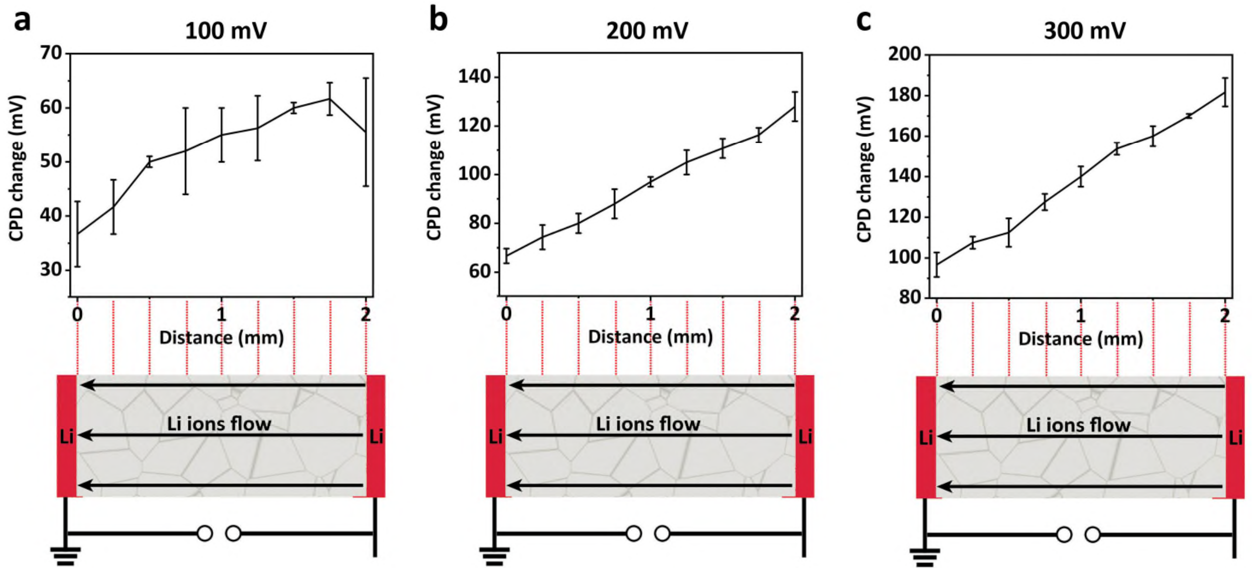

Supplementary Figure 14. Spatial CPD change distribution along LLZO in a symmetrical cell compared with OCV state at different applied potentials. Top row: Local CPD changes relative to the open circuit state, measured at different positions of the LLZO at applied potential s of **a** 100 mV, **b** 200 mV and **c** 300 mV between the Li-WE and Li-CE. The error bars correspond´to three independent experiments. Bottom row: Schematic representation of the cell and the ion flow. The red dotted lines indicate the positions where the CPD values were measured along the LLZO.

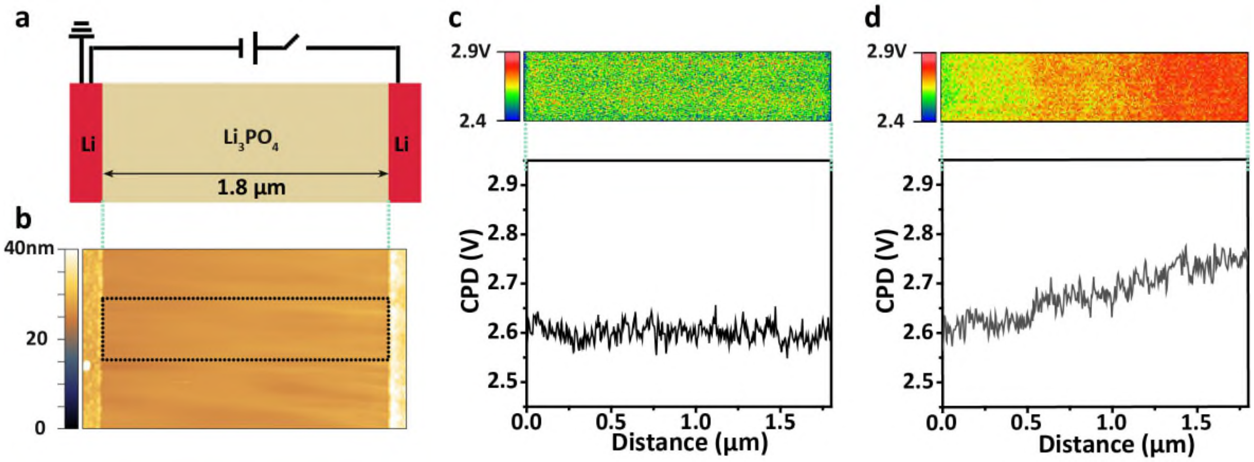

Supplementary Figure 15. KPFM measurement on a amorphous Li_3PO_4 solid electrolyte. a Schematic of Li|Li_3PO_4|Li symmetric cell with Li_3PO_4 amorphous solid electrolyte. The electrode distance is 1.8 µm. **b** topography of the Li|Li_3PO_4|Li symmetric cell cross section. The blue dotted lines indicate the interfaces to Li, respectively. **c** CPD map of the Li_3PO_4 in the region of the dark dotted rectangle in **a** at OCV state (top) and the corresponding averaged CPD line profile along the whole cross section (bottom). **d** CPD map of the Li_3PO_4 in the region of the dark dotted rectangle in **a** at an external potential of 200 mV applied between the electrodes (top) and the averaged CPD line profile along the whole cross section (bottom).

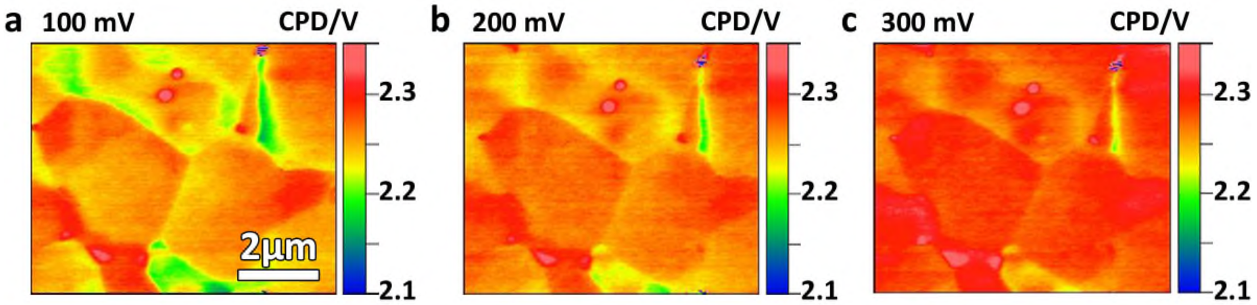

Supplementary Figure 16. CPD maps of LLZO at different applied potentials. CPD map of LLZO at different applied potentials of **a** 100 mV, **b** 200 mV and **c** 300 mV between Li-WE and Li-CE.

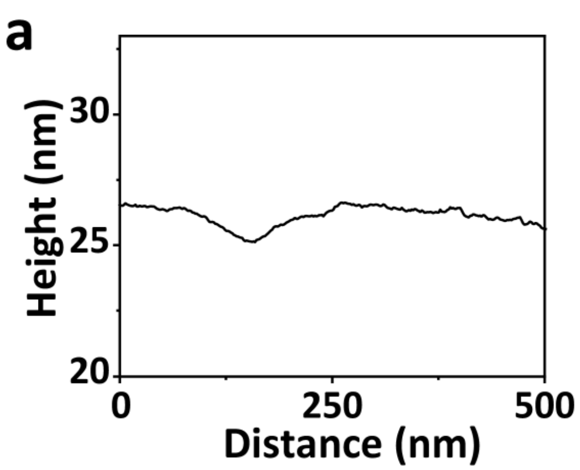

Supplementary Figure 17. Line profiles corresponding to Figure 3 b and c. a Height profile extracted from the dotted line shown Figure 3b. **b** CPD profile extracted from the dotted line in the CPD map of Figure 3c.

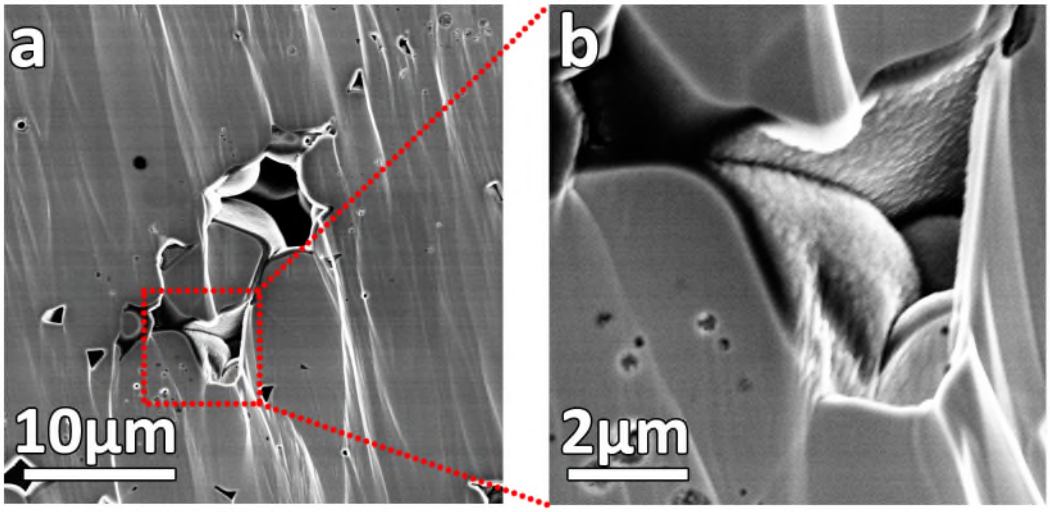

Supplementary Figure 18. Details of Li_2CO_3 at microstructure defects (supplementary for Figure 3d). a Ex-situ SEM images of a LLZO cross section. **b** At a higher magnification, a granular surface was visualized which covered the outer side of grains in LLZO voids or defects. We associate this surface to the presence of Li_2CO_3.

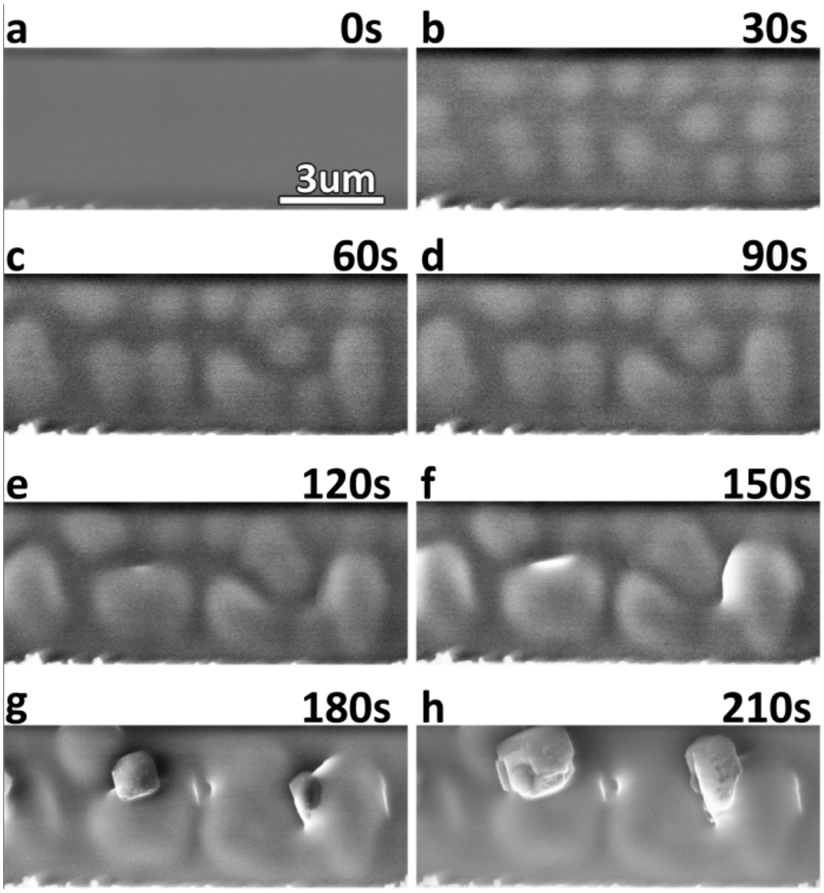

Supplementary Figure 19. Li_3PO_4 surface morpology evolution under electron beam irradiation. Evolution of the Li_3PO_4 amorphous solid electrolyte surface morphology upon electron beam irradiation. The time step between all images is 30 s (from **a** to **h)**.

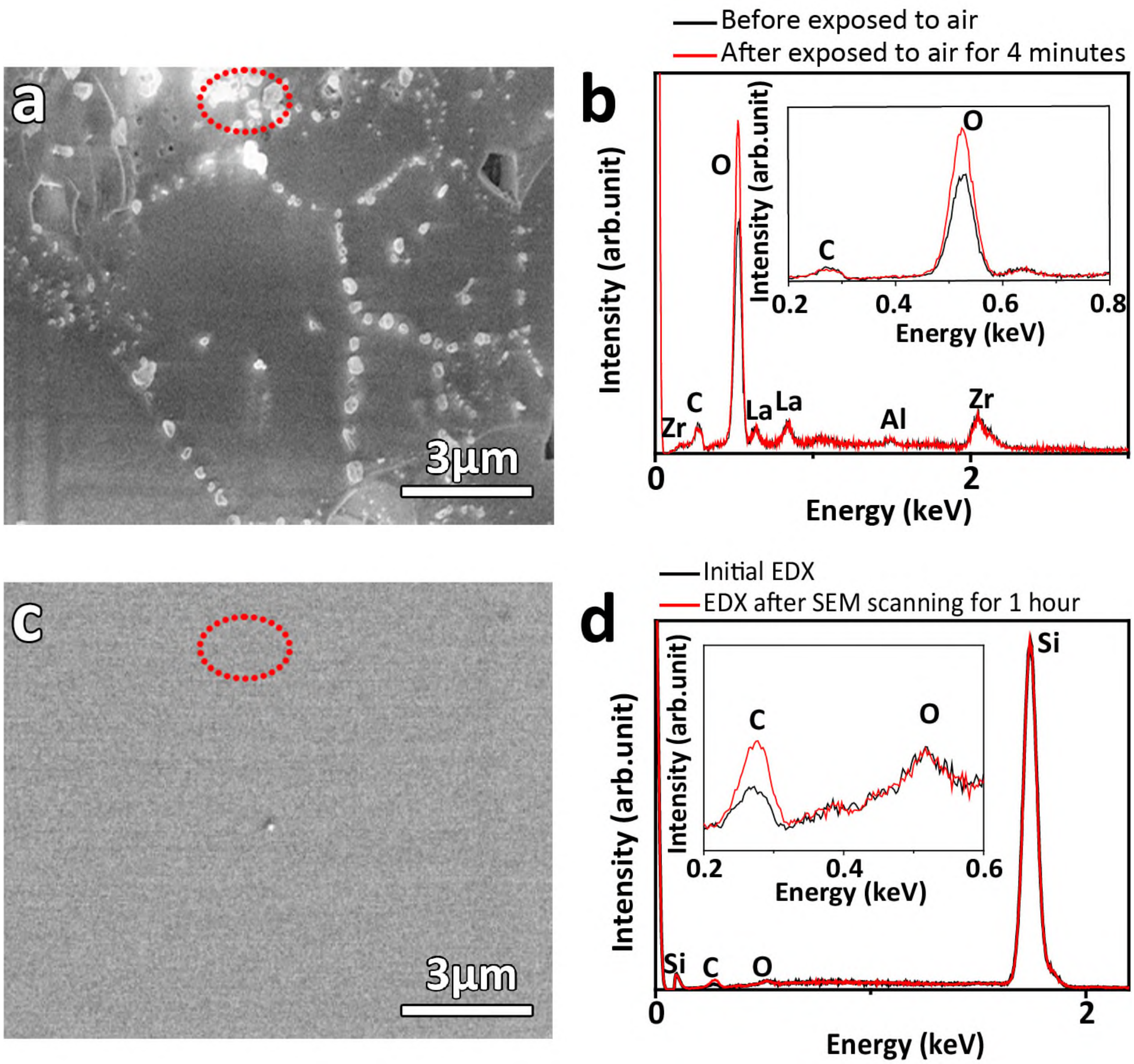

Supplementary Figure 20. Proof for lithium metal expulsions. **a** SEM of a LLZO cross section after irradiation by electrons. Expulsions are visible on the surface. **b** EDX spectra recorded in the region marked by a red dotted circle in **a.** The black line corresponds to the spectrum of the sample before exposure to air and the red line to the one after exposure to air for 4 minutes. The inset shows a higher resolved spectrum around the signal corresponding to oxygen. **c** SEM of a pure silicon wafer as reference. **d** The EDX spectra recorded in the region marked by a red dotted circle in **c.** The black line corresponds to the initial EDX spectrum after a freshly cleaned silicon wafer was put into the SEM. The red line corresponds to the EDX spectrum after an hour of continuous SEM scanning.

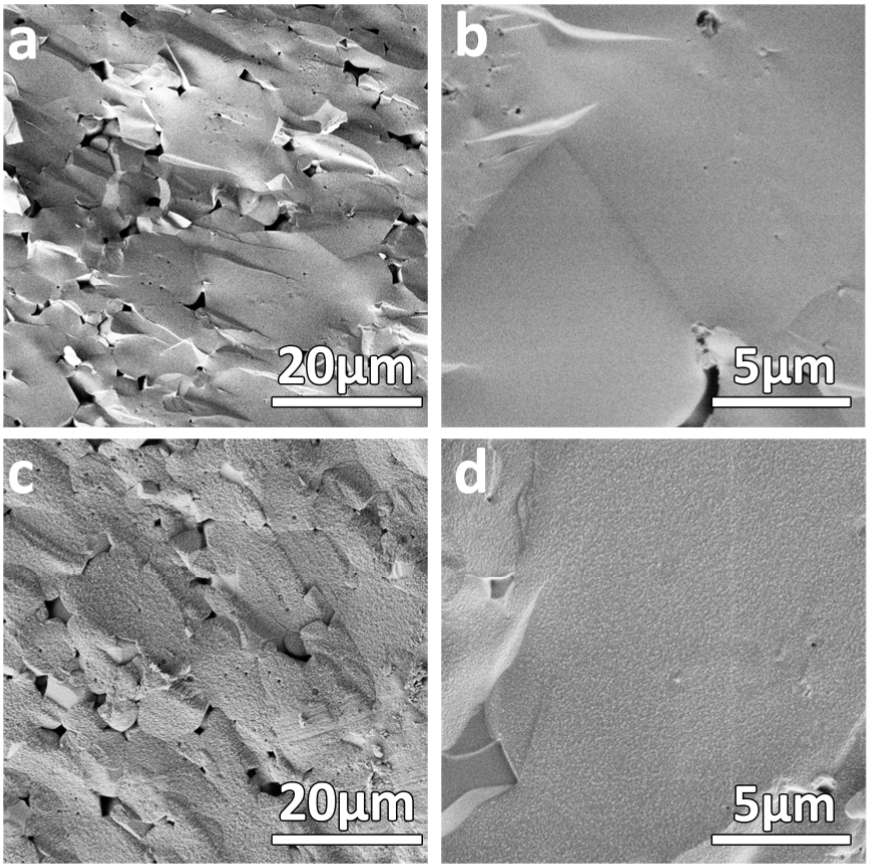

Supplementary Figure 21. Surfaces with and without Li_2CO_3. a and b We prepared a LLZO cross section in an argon environment in order to avoid Li_2CO_3 formation. Then we analyzed the surface with an SEM. **c and d** the same cross section exposed to air for several hours. Surfaces that were not exposed to air are much smoother.

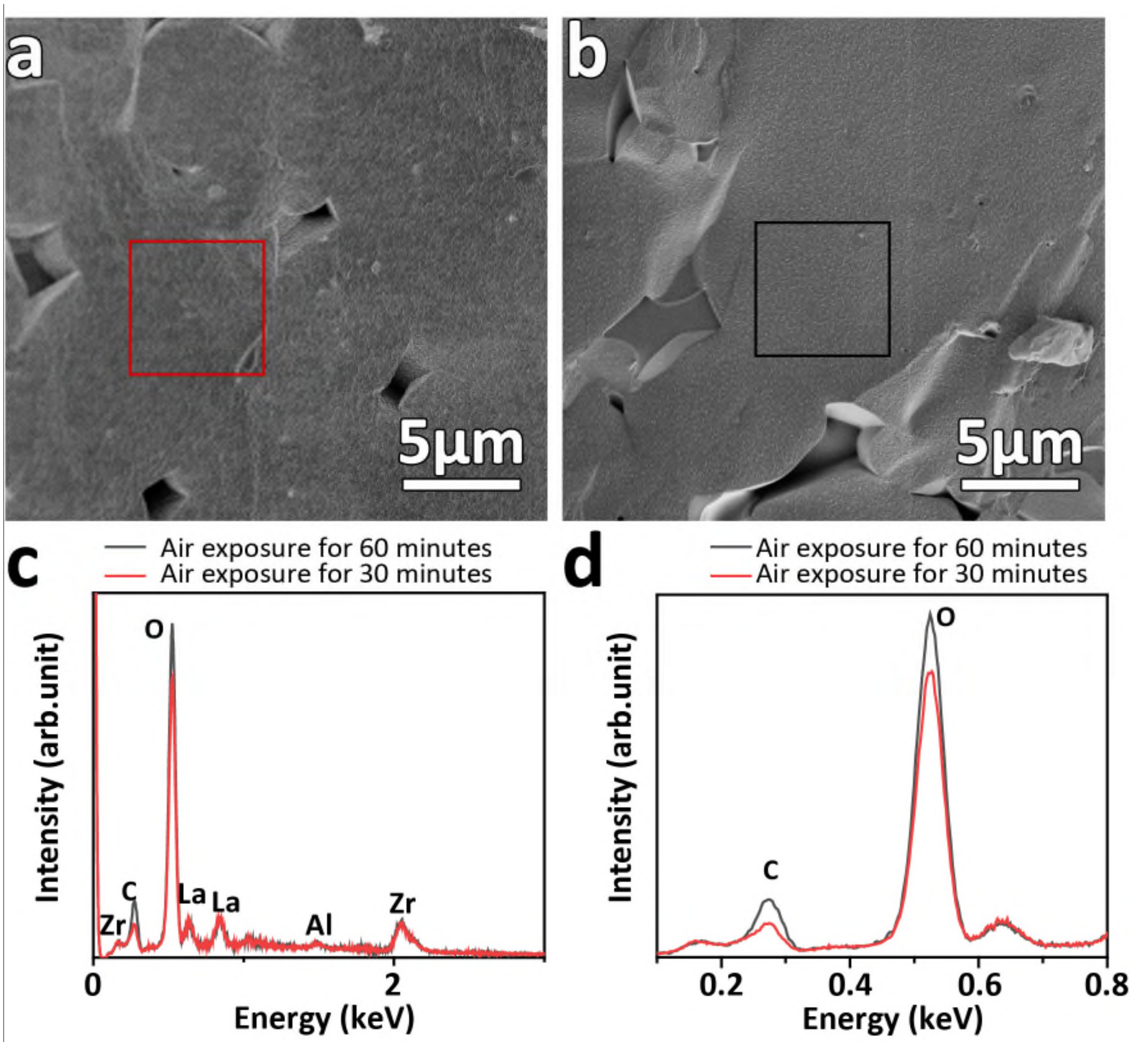

Supplementary Figure 22. Identification of Li_2CO_3 on the LLZO surface. a SEM result of a cross section of LLZO after exposure to air for 30 minutes. **b** The cross-section of LLZO after exposure to air for 60 minutes. **c** EDX spectra of the LLZO surface after exposure to air for 30 minutes (red line) and 60 minutes (black line). Both EDX measurements were acquired in the region marked with squares in **a** and **b**, respectively. **d** Higher resolution EDX spectra of the C and O signals.

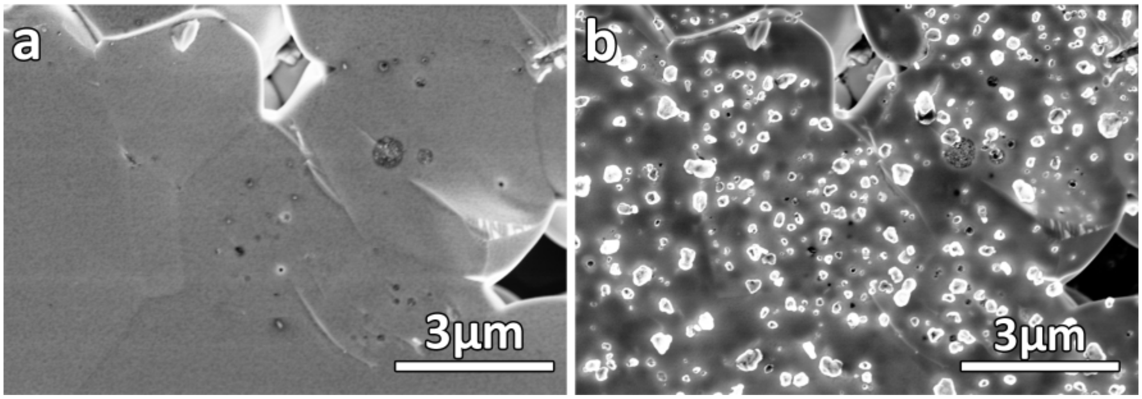

Supplementary Figure 23. The presence of a Li_2CO_3 layer on the LLZO surface affects expulsions at grain boundaries under electron beam irradiation. a LLZO surface was exposed to air for 1 hour. **b** Morphology change of (a) after 5 minutes after electron beam irradiation.

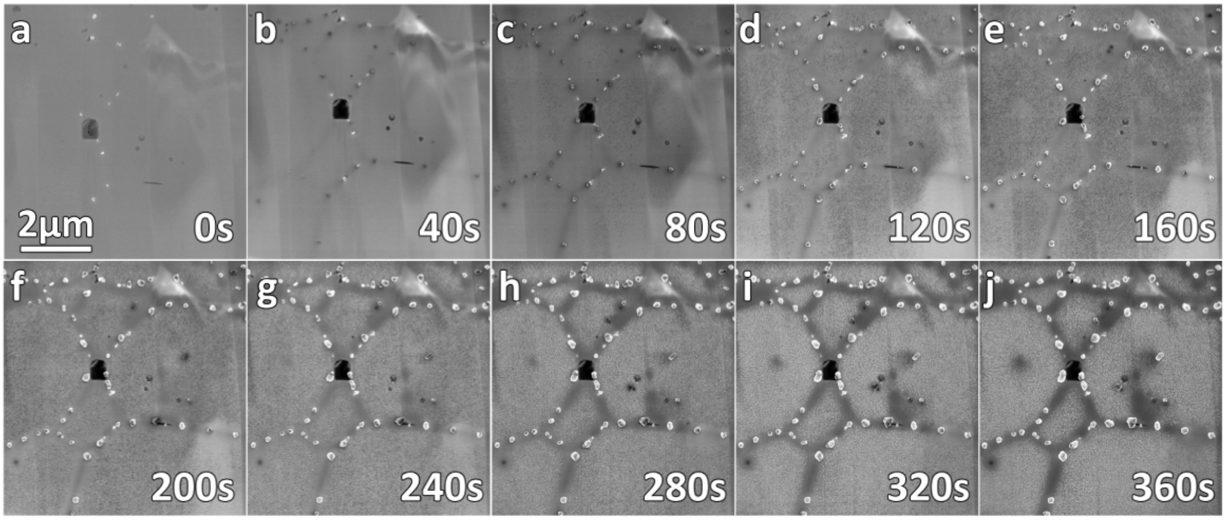

Supplementary Figure 24. Excluding the role of surface roughness on Li expulsion. Study of a LLZO surface which was polished by argon-ion milling. Evolution of the morphology change under electron beam irradiation with increasing time from **a** 0 s to **j** 360 s with time step of 40 s.

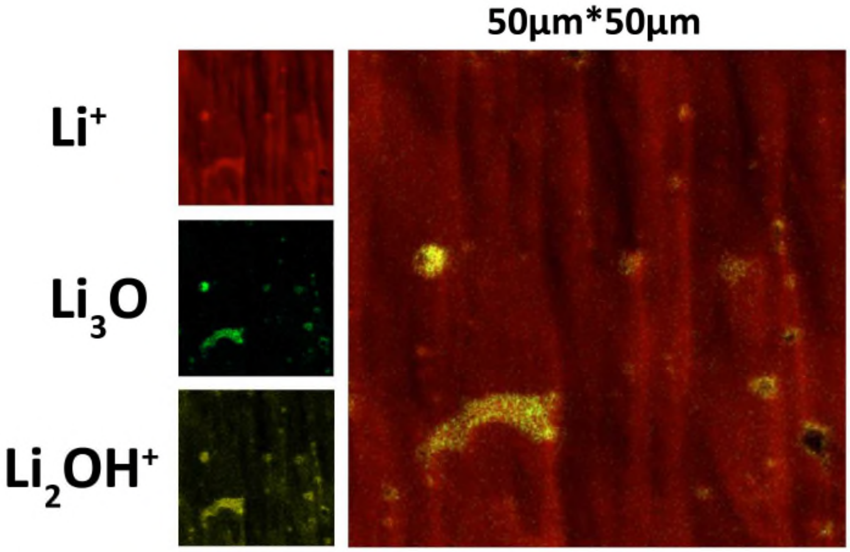

Supplementary Figure 25. Li concentration comparison between grain boundaries and in-grain parts. Ex-situ TOF-SIMS results of the lithium distribution in a LLZO cross section. Brighter colors indicate higher lithium-ion concentration.

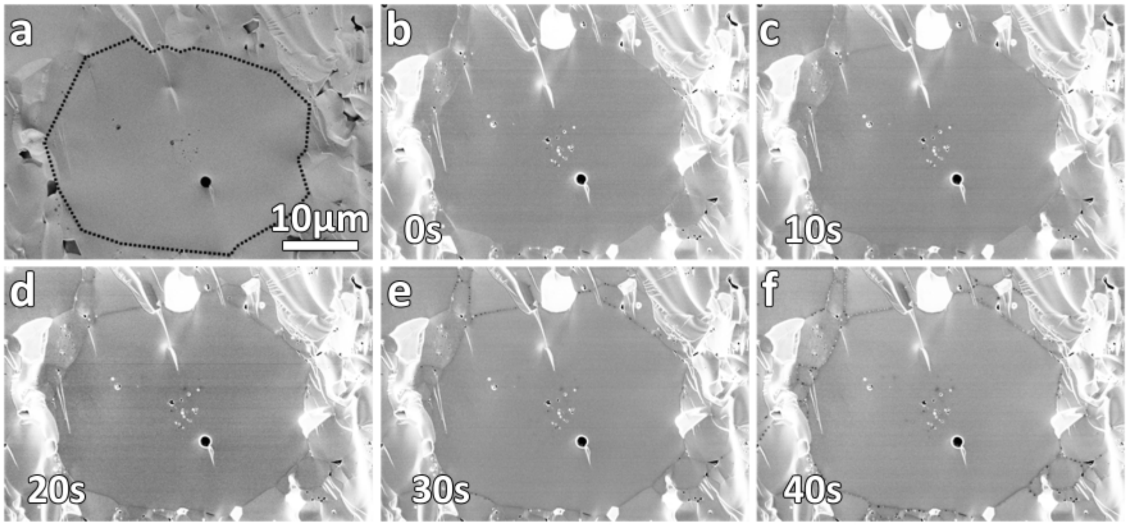

Supplementary Figure 26. Negative charge effect of grain boundaries can be observed at all grain boundaries in large scale. Changes of LLZO grain boundaries in the initial stage of electron beam irradiation of a freshly prepared cross section. A freshly cross section is prepared by breaking a LLZO piece into two piece to expose new cross section in argon filled glovebox.

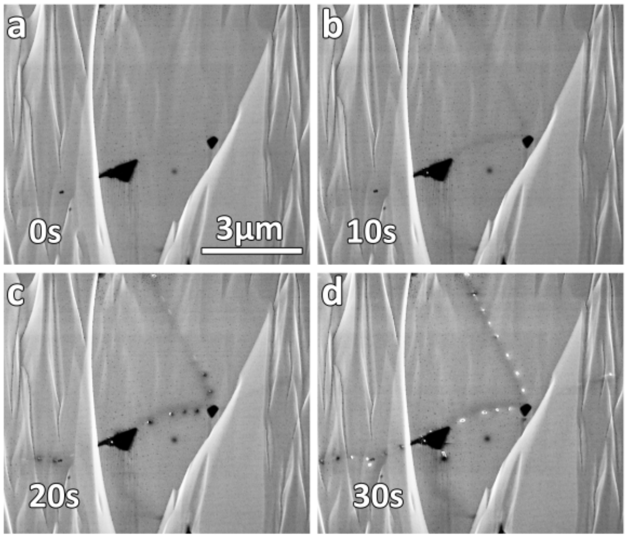

Supplementary Figure 27. Excluding the effect of surface roughness on the negative charge effect at grain boundaries. Changes of LLZO grain boundaries in the initial stage of electron beam irradiation on an argon ion milling polished surface.

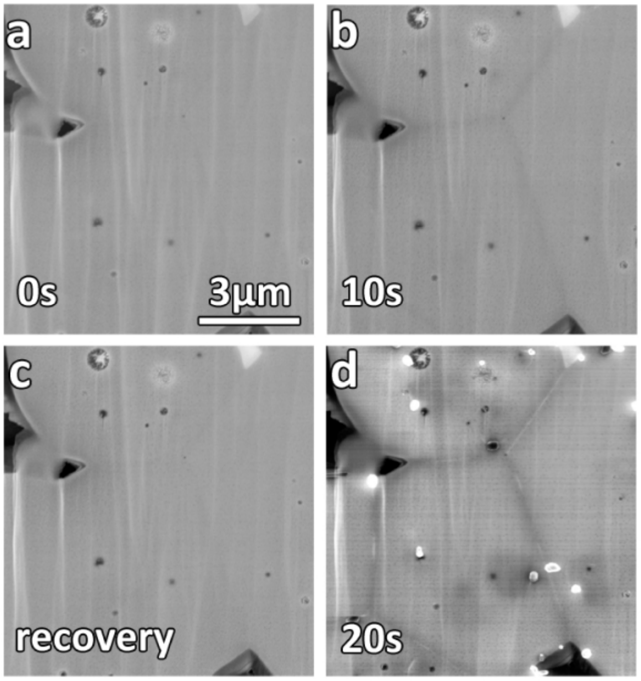

Supplementary Figure 28. The negative charge effect disappears after removing the electron beam in the initial electron beam irradiation stage. Changes of LLZO grain boundaries in the initial stage of electron beam irradiation on an argon ion milling polished surface. **a** The LLZO surface before electron beam irradiation. **b** The same area in **a** after 10 s electron beam irradiation. **c** The same area in **b** after removing the electron beam for half an hour. **d** The same area in **c** after being re-irradiated with the electron beam for 30 s.

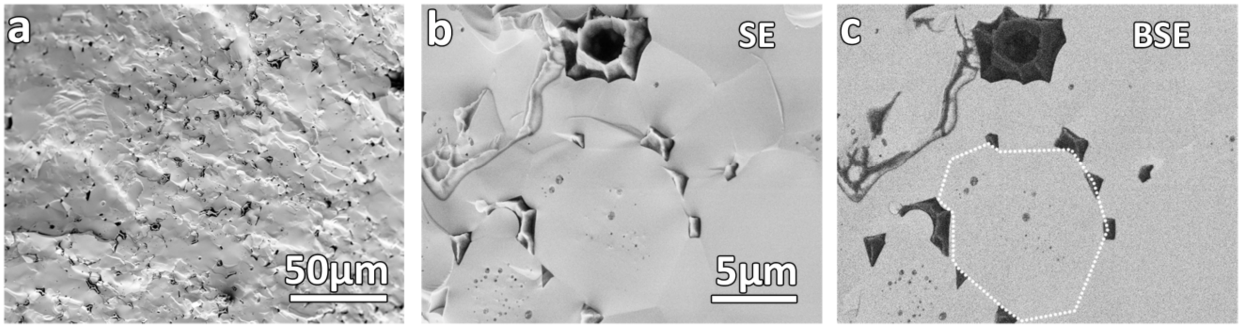

Supplementary Figure 29. Lithium dendrites are able to penetrate grain boundaries. a SEM of a LLZO cross section after lithium dendrites have grown. **b** SE mode SEM of the LLZO cross section with lithium dendrites filing voids and penetrating grain boundaries. **c** BSE mode SEM of the LLZO in the same area with **b**.

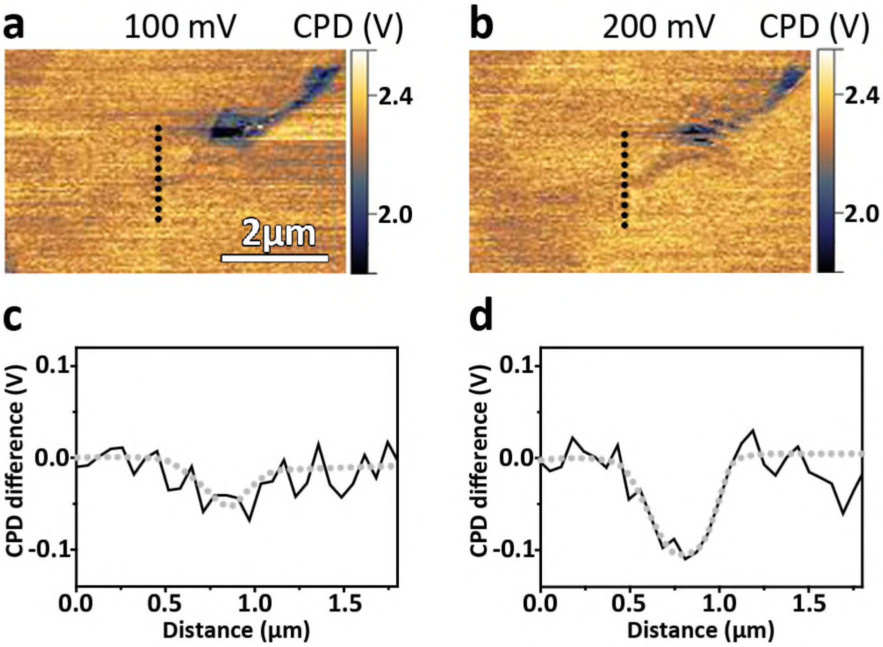

Supplementary Figure 30. A higher applied potential leads to a larger CPD decrease at grain boundaries. We performed an additional CPD measurement of the same LLZO region with Figure 1**c** and Supplementary Figure 13 under different external current density applied to a Li|LLZO|Li symmetric cell. **a** at 0.1 mA/cm^2 (corresponding to 100 mV external potential applied) and **b** at 0.25 mA/cm^2 (corresponding to 200 mV external potential applied). The CPD maps were analyzed by profiles which are outlined by a black dotted line in **a** and **b**. **c** and **d** CPD difference profiles along the dotted lines, respectively. The CPD differences were calculated by subtraction of the CPD value measured under the OCV state.

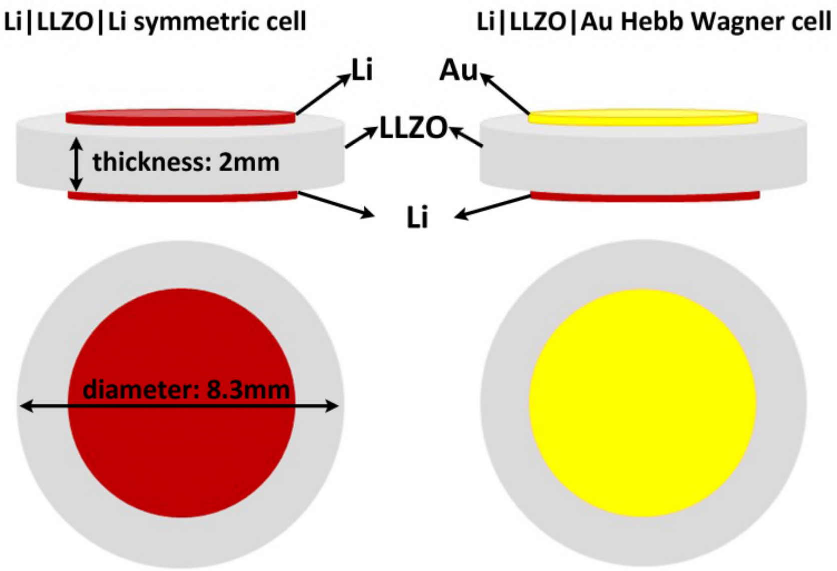

Supplementary Figure 31. Structure of different cells. Schematic structure of a Li|LLZO|Li symmetric cell and a Li|LLZO|Au Hebb Wagner cell.

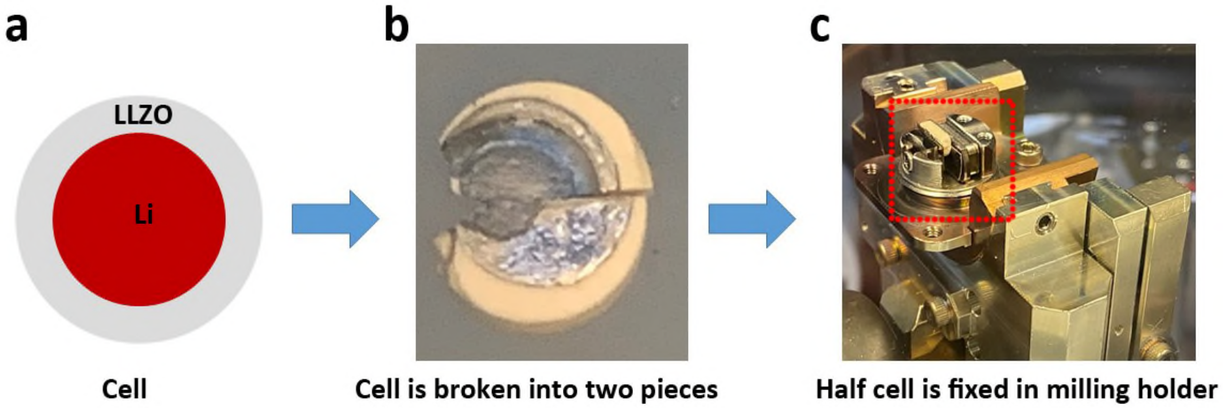

Supplementary Figure 32. Process of preparing cell cross-section. a Schematic structure of prepared cell. **b** Photograph of a cell broken into two pieces. **c** Photograph of a broken cell which is fixed in the cross section milling holder. The part marked with red dotted square is the position where cell is clamped.

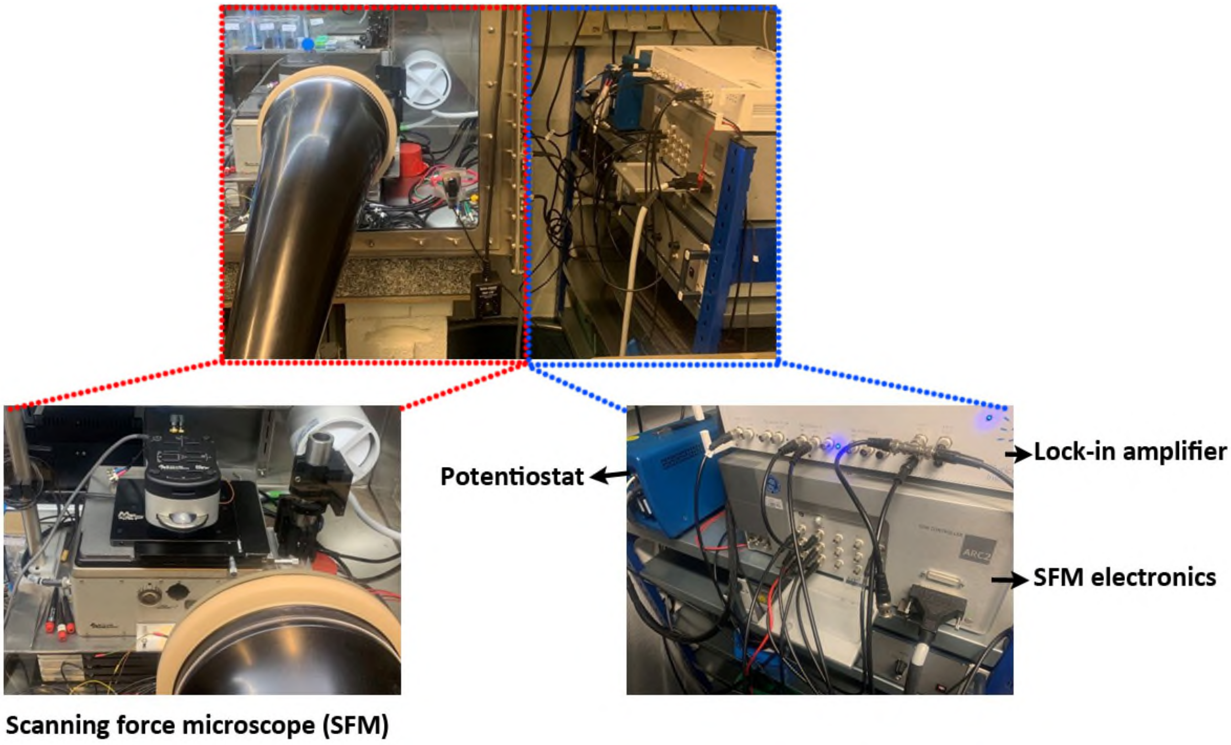

Supplementary Figure 33. Photographs of the operando KFPM/tr-EFM equipment. The red dotted area marks the scanning force microscope (SFM) which is used for operando KPFM and tr-EFM measurements. The SFM is installed in a Ar filled glove box. The blue dotted area shows the SFM electronics, the lock-in amplifier and the potentiostat.

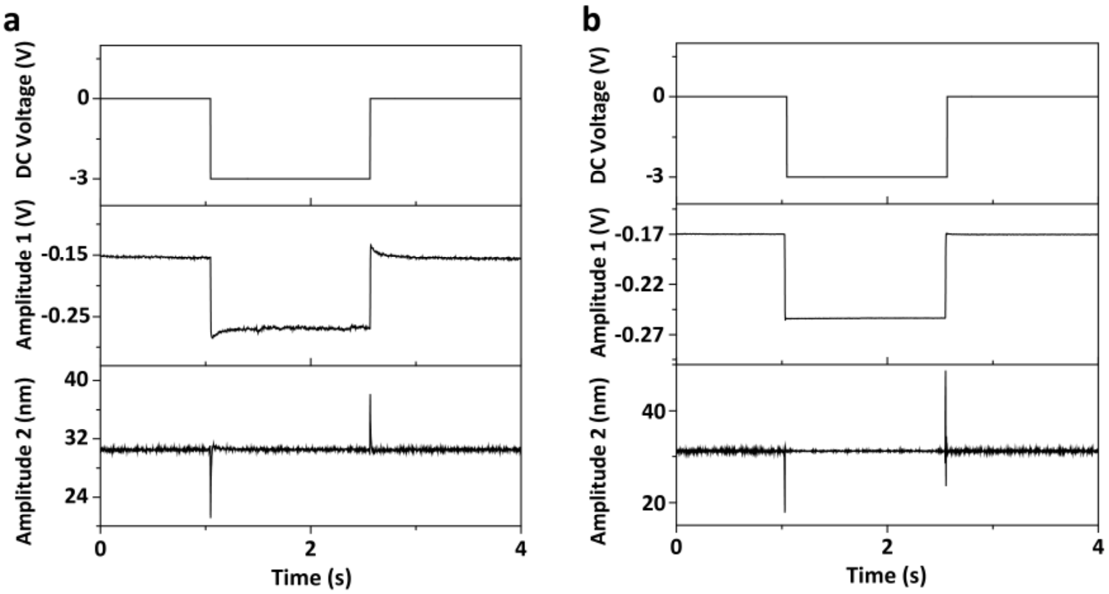

Supplementary Figure 34. Comparison of tr-EFM when applied to LLZO and Au. At each pixel of an image, we applied a DC-voltage for 1.5 seconds with an amplitude of -3 V to the SFM tip in respect to the sample. The corresponding time resolved EFM signals recorded at the first (amplitude 2) and second resonance frequency (amplitude 1) of the cantilever. **a** Measurement on the LLZO and **b** on a gold reference sample.

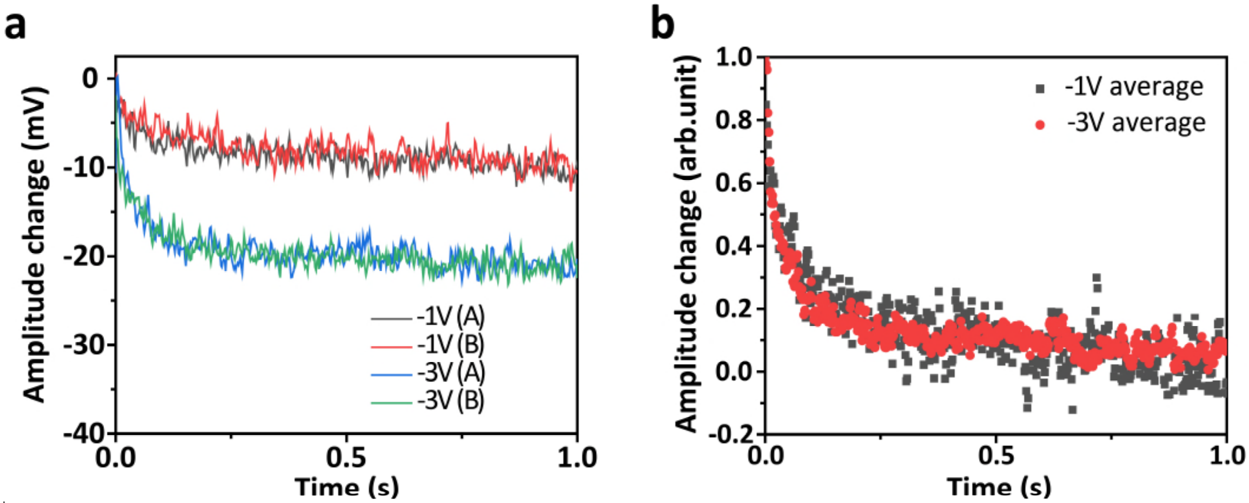

Supplementary Figure 35. Tr-EFM results are independent from the applied DC voltage to the tip. Changes in amplitude 1 corresponding to the tr-EFM signal on LLZO surface different point A and B. **a** We applied different DC bias voltages of -1 V and -3 V to the SFM-tip. **b** The averaged signal on point A and B as shown in (a) after normalization of amplitude 1.

Supplementary Table 1. EIS fitting results of Li|LLZO|Li symmetric cell. In the fitting results, R represents resistance, C represents capacitance and α represents constant phase exponent for capacitance.

	bulk	grain boundary
R / Ω cm^2	225.19 ± 0.74	61.00 ± 0.83
$C(Q)$ / F	$(4.35 \pm 0.22) \cdot 10^{-11}$	$(1.17 \pm 0.13) \cdot 10^{-8}$
α	0.938 ± 0.003	0.826 ± 0.010

Supplementary Note 1

Argon ion milling and evaluation of its effects

KPFM was performed on a freshly prepared LLZO cross section, which was not polished by argon-ion milling. A freshly prepared cross section means LLZO solid electrolyte or Li|LLZO|Li symmetric cell is broken into two piece to expose fresh cross section in argon filled glovebox.

The in-grain parts are relatively flat and the CPD value was measured to be around 2.3 V (Supplementary Figure 5). This value is similar to the CPD value for the LLZO surface after polishing with argon ions in Figure 1c. This result shows that argon ion milling does not drastically change the LLZO surface. For a freshly prepared LLZO surface, grain boundaries show a significant crosstalk between topography and CPD (Supplementary Figure 5). Here, argon-ion milling is a useful method to effectively decrease roughness. The latter reduces CPD crosstalk especially at grain boundaries.

Supplementary Note 2

KPFM measurement on a Li electrode having a passivation layer

The lithium metal surface is covered by a passivation layer, consisting of Li_2CO_3, LiOH and Li_2O.[2] Recently, Otto et al. showed that such passivation layers with a thickness ranging from several nm to hundreds of nm on a lithium metal surface are present even for lithium metal that was stored in glove boxes filled with argon gas. This passivation layer is ion-conducting but its ionic conductivity is much lower than of LLZO.[3]

When a constant current was applied to a Li|LLZO|Li symmetric cell, lithium ions were extracted from the Li-WE and deposited at the Li-CE. At the Li-CE|LLZO interface, lithium ions move from LLZO and enter into the passivation layer because it is ion-conducting. But in the passivation layer the lithium ion conductivity is lower compared to metallic Li. Therefore lithium ions in the passivation layer are reduced into metallic lithium at the passivation layer/Li-CE interface close to the Li-CE/LLZO interface. This effect causes a higher lithium ion concentration in the passivation layer close to LLZO (Supplementary Figure 8a). Thus, a CPD increase can be observed at the Li-CE surface close to the Li-CE|LLZO interface even though the Li-CE is connected to ground.

At the Li-WE side, the Fermi level of Li-WE and its surface passivation layer shift with the external potential. At the same time, lithium ions move across the passivation layer to LLZO. In addition, some lithium ions from the oxidized metal lithium move into the passivation layer by crossing the passivation layer|Li-WE interface (Supplementary Figure 8b). Thus, the concentration of lithium ions in the passivation layer almost does not change. As a result, we observe an overall uniform CPD increase on the Li-WE surface.

Supplementary Note 3

KPFM measurement on Li|LLZO|Au Hebb-Wagner cell

KPFM is a technique widely used to measure the work function of metals and semiconductors, which belongs to the inherent electronic properties of materials. Typically, samples are only electrically connected to the KPFM electronic circuitry. In our case, the sample is additionally part of another electric circuit which charges and discharges the device via a potentiostat. In addition, LLZO is an ionic conductor with negligible electronic conductivity. Therefore, the physical meaning of the KPFM signal change needs to be clarified.

From a electrochemical view, KPFM traditionally measures the Volta potential ψ of metallic and semiconductor samples.[4] In case the sample is only connected to the KPFM circuit, the KPFM-tip has the same electrochemical potential of electrons as the sample ($\tilde{\mu}_{\mathrm{e}^-,\mathrm{tip}} = \tilde{\mu}_{e^-,sample}$). Now, the CPD value can be written:

$$CPD = \psi_{\mathrm{sample}} - \psi_{\mathrm{tip}} = \frac{\Phi_{\mathrm{sample}} - \Phi_{\mathrm{tip}}}{-e} \qquad \text{(S1)}$$

Φ_{sample} and Φ_{tip} are the work functions of sample and KPFM tip, respectively. Therefore, the CPD signal measured by KPFM equals to the work function difference between sample and KPFM-tip.

In a Li|LLZO|Li symmetrical cell, the dominating charge carrier in the electrolyte LLZO are the mobile lithium ions. Thus, equation S1 does not apply. In this case, the CPD corresponds to the Volta potential difference between the sample and the tip. Consequently, if an external potential is applied along the cell, the CPD change will be related to the Volta potential change of the sample (ψ_{sample}).

The inner potential/Galvani potential (ϕ) is given by the sum of the Volta potential (ψ) and the surface potential (χ)

$$\phi = \psi + \chi \qquad \text{(S2)}$$

For LLZO, the surface potential χ, i.e., the dipole nature of the surface, is constant beyond a length scale longer than the unit cell when a lithium ion current flows along LLZO. LLZO is in a steady state with no spatial changes in surface potential χ. In other words, the composition and work function change of LLZO do not vary locally in case of a symmetric cell. Thus, measurements of changes in the CPD correspond exclusively to changes of the inner/Galvani potential ϕ. We like to emphasize that KPFM offers a unique perspectives for contact-free measurements of Galvani potential “landscapes”.

In order to prove experimentally that the CPD signal measured by KPFM on LLZO is related to the inner potential ϕ, we performed measurements on LLZO, yet in this case in an asymmetric Li|LLZO|Au Hebb-Wagner cell. Without an externally applied potential to a Li|LLZO|Au Hebb-Wagner cell, the inner potential ϕ is constant across the whole LLZO solid electrolyte (Supplementary Figure 11a). Here we do not consider interface effects at the electrodes, respectively. When a positive external DC voltage is applied to the gold electrode and the lithium electrode is connected to ground, the cell polarizes. Then the external electrical potential in LLZO is shielded because gold does not provide lithium ions. There is no ion flux in LLZO in the steady state. Apart from the interfaces to the electrodes, the LLZO is unchanged in composition. Thus, the inner potential ϕ of LLZO remains constant (Supplementary Figure 11b). However, electrons move along LLZO due to the chemical potential difference of electrons in the polarized solid electrolyte.[5,6]

When a positive DC voltage is applied to the lithium electrode and the gold electrode is connected to ground (Supplementary Figure 11c), lithium ions continuously move from the lithium electrode to the gold electrode. In this situation, ϕ is expected to show a linear decrease from the lithium electrode to the gold electrode.

In the beginning of the experiment under ion-blocking conditions (Supplementary Figure 12a), when a DC voltage of 500 mV was applied to the gold electrode, a polarization current of around 87 µA flows initially across the whole cell. The current decreased to around 4.5 µA after 10 s. Then after 100 s, the current remained stable at a value around 30 – 40 nA (Supplementary Figure 12b). For this cell and this DC voltage, ions move from the side of the ion-blocking electrode (Au) to the side of the lithium electrode within the first few seconds. Gold, as the ion-blocking electrode, cannot provide lithium ions to the solid electrolyte. Thus, the solid electrolyte polarizes and shields the Galvani potential in the solid electrolyte, finally leading to a vanishing ionic current. However, electrons are still moving due to chemical potential difference of electrons in the polarized solid electrolyte.[5,6] Therefore, in the first 100 s after switching on the DC voltage the current arises from ions and electrons, while afterwards the current reflects the electron current only. Directly after switching on the DC voltage, we measured at point A a CPD value increasing by 180 mV. At point B, which is located closer to the gold electrode, the CPD value increased by 320 mV (Supplementary Figure 12c - d). Thus, both points reveal the local magnitude of the external electrical potential that was applied and directly after ions started to move. Then, during the first 100 s, the CPD change compared to CPD value of the pristine state decreased at point A and B to 0, respectively. In particular, after 100 s, there was almost no CPD change difference between point A and point B within the accuracy of the measurement.

Then, a reverse DC voltage of 500 mV was applied, i.e., a positive potential to the lithium electrode (Supplementary Figure 12e). Now, the current measurement showed a current flow of around 0.5 mA in the beginning (Supplementary Figure 12f). The current increased up to 0.6 mA after 400 s, probably due to morphological/chemical changes of the electrodes. At the same time, the color of the gold electrode in the cell changed gradually to dark silver, which clearly indicated the formation of a Li_xAu alloy. The reason for the current is a continuous lithium ion flow from the lithium electrode to the gold electrode. We attribute the current increase to a decrease in interface resistance owing to formation of the Li_xAu alloy. The latter promoted a better electrical contact with the LLZO. Directly after switching on the DC voltage, we measured an increased CPD value at point A, which is located closer to the lithium electrode of 300 mV. At point B the increase of CPD value was around 125 mV (Supplementary Figure 12g - h). Then, afterwards for both points the CPD values slowly increased by 20 – 30 mV after 400 s. We repeated these measurements with newly prepared pristine cells several times and always found a similar behavior.

Additionally, we applied different DC voltages to the gold electrode (100 mV, 500 mV, 1000 mV and 2000 mV) of Li|LLZO|Au Hebb-Wagner cells. After reaching a stable polarization state, the electronic current across the whole cell was below 50 nA for all applied DC voltages. The corresponding CPD measurements on LLZO at different distances from the lithium electrode showed all values $< |10|$mV and no systematic variation (Supplementary Figure 13). Within the resolution of our experiment, the Galvani potential along LLZO is constant at all applied potentials.

Both experiments provide a direct proof that the KPFM signal for solid electrolytes corresponds to the Galvani potential rather than the (electronic) work function. We like to note that such proof has not been reported before in the literature.

Supplementary Note 4

KPFM measurement on a Li_3PO_4 amorphous solid electrolyte

For comparison, we performed KPFM measurements on a Li|Li_3PO_4|Li symmetric cell (Supplementary Figure 14). The CPD of the Li_3PO_4 solid electrolyte reveals no grain boundaries or secondary phases in the amorphous Li_3PO_4 solid electrolyte. Furthermore, when an external potential is applied to the Li|Li_3PO_4|Li symmetric cell the electrical potential gradient in the Li_3PO_4 solid electrolyte is similar to the one measured in the LLZO solid electrolyte. Due to the absence of grain boundaries, there are no sharp localized electrical potential drops in the Li_3PO_4 solid electrolyte. We measured a rather constant potential gradient.

Supplementary Note 5

Evidence of expulsions on LLZO under electron beam irradiation are metallic lithium

EDX cannot detect signals from metallic lithium. In order to demonstrate that the expulsions are pure lithium metal, we first recorded an EDX spectrum of the expulsed particles. Then, we vented the SEM vacuum chamber and exposed the sample to air for 4 minutes. Under these conditions, metallic lithium reacts with O_2 and forms lithium oxide. In addition, we evacuated the SEM chamber again and recorded another EDX spectrum at the same position. Now, the intensity of the O peak increased significantly while the signal intensity of other elements remained the same (Supplementary Figure 20b). Therefore, we conclude that the expulsions correspond to metallic lithium, which subsequently can react with O_2 from air.

In order to explore the reason for the presence of a C signal on the fresh LLZO surface, we recorded an EDX spectrum on a silicon wafer out of its original transportation box. In this case, minor C and O signals are present. The O signal results from the formation of silicon oxide on the surface which is present after cleaning. Then we continuously scanned with the electron beam for around 1 hour. Subsequently, we recorded an EDX spectrum at the same place. The Si and O signal did not change but the C signal increased (Supplementary Figure 20d). Thus, we conclude that the C signal originates from small amounts of carbonaceous compounds remaining in the atmosphere of the SEM chamber but not from LLZO.

Supplementary Note 6

Effect on electron beam irradiation experiment when LLZO surface has oxidation layer

For the lithium expulsion experiment under electron beam irradiation we prepared all cross sections in an argon environment in order to avoid Li_2CO_3 formation.[7] SEM images of LLZO cross section before and after exposed to air revealed that freshly broken surfaces were much smoother and do not exhibit small features (Supplementary Figure 21).

The SEM analysis of a LLZO cross section after exposure to air for 30 min and 60 min revealed that the surface morphology of the by-products formed by LLZO reaction with air changed from seeds to particles. The corresponding EDX spectra show that only the C and O peak intensities increased, while those of La, Zr and Al remained constant (Supplementary Figure 22). The latter is in agreement with the formation of Li_2CO_3 on the LLZO surface. In particular, the morphology of the Li_2CO_3 cover layer on LLZO is similar to results reported by Xia et al.[8]

Lithium expulsions are detected for LLZO surface covered by a Li_2CO_3 layer upon electron beam irradiation (Supplementary Figure 23a), too.[7] In this case, the whole Li_2CO_3 layer is uniformly covered with expulsions and distinct areas with more or larger expulsions, e.g. at grain boundaries, are not observed (Supplementary Figure 23b).

Supplementary Note 7

TOF-SIMS: Grain boundaries do not show a different Li concentration compared to in-grain regions.

For ToF-SIMS measurements the surface should be as smooth as possible. Therefore, the surfaces of LLZO cross sections were polished by our argon-ion milling procedure. On such samples, we performed TOF-SIMS measurements at an area of 50 µm × 50 µm (Supplementary Figure 25). The diameter of LLZO grains is in the range of 3 – 7 µm. Therefore, the measured area contains over hundreds of grains and many grain boundaries. The Li^+ signal indicates round features with a diameter of 1 – 3 µm. Those regions could be inner defects in grains. However, those round features do not overlap with the typical grain boundary distribution in LLZO. Therefore, there is no obvious lithium concentration difference between grain boundaries and in-grain parts in LLZO.

Interestingly, we observed that a higher Li_3O and Li_2OH^+ signal appears at the edge of voids. Grains that were not fused together in the sintering process caused these voids. Consequently, the strong Li_3O and Li_2OH^+ signals arise from the reaction of LLZO with H_2O and CO_2 in air during sintering.[9]

Supplementary Note 8

Negative charge effect of grain boundaries

For the LLZO surface in Supplementary Figure 28a, we did not observe any contrast between grain boundaries and in-grain parts. After electron beam irradiation for 10 s, grain boundaries became darker compared to in-grain parts. Then, we switched the electron beam off for half an hour. The subsequent SEM image shows that the color contrast at grain boundaries almost completely disappeared (Supplementary Figure 28c). After a subsequent electron irradiation on the same area again for 20 s, the grain boundaries become darker again. Then, also lithium expulsions are visible.

The strong decrease in contrast at grain boundaries before lithium expulsion demonstrates that the initial contrast at grain boundaries in the initial electron beam irradiation stage is related to electron beam irradiation and not to irreversible changes at grain boundaries.

Supplementary Note 9

Theory of KPFM measurements

In our heterodyne KPFM measurement the topography is imaged using the first resonance frequency of the cantilever. Non-linear electrostatic interactions imposed at frequency ω_E of the tip with the sample surface lead to the appearance of sidebands at ω_m+/- ω_E. The electrostatic force on the tip $F_{\omega_m \pm \omega_E}$, can now be described by[10,11]

$$F_{\omega_m \pm \omega_E} = -A_m C''(V_{dc} - V_{CPD})V_{ac}[\sin\big((\omega_m - \omega_E)t\big) + \sin\big((\omega_m + \omega_E)t\big)] \quad \text{(S3)}$$

Where A_m is the mechanical oscillation amplitude, C'' is the electrostatic force gradient. In particular, we set ω_E to the difference between the second and first resonance frequency. Then the electrostatic force is amplified by the second mechanical resonance frequency. When $V_{dc} = V_{CPD}$, $F_{\omega_m \pm \omega_E}$ becomes 0. The electronic feedback circuit adjust for a minimum force at $\omega_m \pm \omega_E$ by adjusting V_{dc}. Consequently, V_{dc} is the measured value for V_{CPD}.

Supplementary Note 10

Theory of tr-EFM measurements

In tr-EFM measurements, an AC voltage was applied to generate electrostatic forces between the tip and the sample surface. For the AC bias, we used the second resonance frequency of the cantilever (ω_2) in order to avoid interference between the electrostatic force amplitude and the mechanical amplitude at first resonance frequency that was used for topography imaging of the surface.

In tr-EFM measurements, an electrostatic force ($F_{es(t)}$) is induced by an AC bias voltage ($V_{ac}\sin(\omega_2 t)$):[12,13]

$$F_{es(t)} = \frac{1}{2}\frac{\partial C_{(z,t)}}{\partial z}\Delta V(t)^2 \quad \text{(S4)}$$

$$F_{es(t)} = \frac{1}{2}\frac{\partial C_{(z,t)}}{\partial z}[V_{CPD} - V_{ac}\sin(\omega_2 t)]^2 \quad \text{(S5)}$$

$$F_{es(t,\omega_2)} = \frac{\partial C_{(z,t)}}{\partial z}V_{CPD}V_{ac}\sin(\omega_2 t) \quad \text{(S6)}$$

where C is the tip-sample capacitance, z is the direction normal to the sample surface, $\frac{\partial C_{(z,t)}}{\partial z}$ is the gradient of the capacitance between cantilever tip and measured LLZO surface and V_{CPD} is the contact potential difference between sample and tip.

In addition, at each measurement point, a DC voltage (V_{dc}) was applied to the tip. The DC voltage drives ions in the LLZO towards the tip which creates a time dependent internal potential $V_{in(t)}$. After the DC voltage is decreased to 0, mobile ions return to an equilibrium stage. The movement of ions alters the electrostatic force between tip and the LLZO surface. This internal potential changes the gradient of capacitance $\frac{\partial C}{\partial z}$ owing to the time dependent change of Li ion concentration. Then the electrostatic force changes to:

$$F_{es(t,\omega_2)} = \frac{\partial C_{(z,t)}}{\partial z}(V_{CPD} + V_{in(t)} - V_{dc})V_{ac}\sin(\omega_2 t) \quad \text{(S7)}$$

When the DC voltage of the tip is decreased to 0, then the time dependent electrostatic force changes to:

$$F_{es(t,\omega_2)} = \frac{\partial C_{(z,t)}}{\partial z}(V_{CPD} + V_{in(t)})V_{ac}\sin(\omega_2 t) \quad \text{(S8)}$$

In particular, the internal potential $V_{in(t)}$ and the gradient of capacitance $\frac{\partial C_{(z,t)}}{\partial z}$ changes when Li ions move to an equilibrium position. Both parameters follow the same time-dependence. We operated the tr-EFM by first applying a DC potential of -3 V for 1.5 seconds to the tip and then switching it to zero. During the latter switch, the time dependent electrostatic force was analyzed by following amplitude changes of the resonance frequency $A_{(t,\omega_{f2})}$

$$A_{(t,\omega_2)} \sim \frac{F_{es(t,\omega_2)}}{k} \quad \text{(S9)}$$

The vibrational amplitude changes $A_{(t,\omega_2)}$ reflect ion concentration changes at different measurement points on LLZO. This tr-EFM procedure has been used to compare differences in ion diffusion in different regions in ionic conductors.[14-16]

In order to record a tr-EFM signal, we applied an AC potential with an amplitude of 1 V at a frequency corresponding to the second resonance frequency of the cantilever. In addition, we applied a rectangular DC voltage with an amplitude of -3 V and a duration of 1.5 s to the tip. In order to detect electrostatic forces induced by the AC potential, we recorded the amplitude of oscillation at a frequency corresponding to the second resonance frequency of the cantilever (amplitude 1) using a Zurich Lock-In amplifier. In addition, we plotted the amplitude of the cantilever oscillation at the first resonance frequency of the cantilever (amplitude 2). This amplitude is used for the topography feedback electronics and is excited by the piezoelectric actuator. In this case, the feedback electronics regulate the z-position of the piezoelectric element to keep an amplitude around 30 nm. Upon applying the DC voltage, the electronic feedback loop regulates within 40-50 ms the z-position of the piezoelectric element and compensates for the electrostatic forces acting between the entire cantilever and the sample. The remaining slower changes of amplitude 1 arise from ion movement in LLZO (Supplementary Figure 31a).

We performed reference measurements on a pure gold sample, which is not ion conducting. Correspondingly, we did not observe a slow change of amplitude 1 (Supplementary Figure 31b).

In order to probe the dependence on the magnitude of the DC-voltage on the ion diffusion time constant, we normalize the amplitude shift vs. time curve. We defined the average stable amplitude change after removing the DC-voltage as A. Then for each point on the curve, the amplitude change (arb.unit) equals:

$$\text{normalized amplitude (arb. unit)} = \frac{A'(t)-A}{|\mathrm{A}|} \quad \text{(S10)}$$

Where A' is the measured time dependent amplitude shift. No significant difference between the signal recorded for a DC-voltage of -1 V and -3 V was present after normalization of the amplitude (Supplementary Figure 32). Therefore, the fitted time constant was not affected by the magnitude of the applied DC-voltage.

Supplementary Reference List

1 Krauskopf, T., Hartmann, H., Zeier, W. G. & Janek, J. Toward a fundamental understanding of the lithium metal anode in solid-state batteries—an electrochemo-mechanical study on the garnet-type solid electrolyte $Li_{6.25}Al_{0.25}La_3Zr_2O_{12}$. *ACS Appl. Mater. Interfaces* **11**, 14463-14477 (2019).

2 Naudin, C. et al. Characterization of the lithium surface by infrared and Raman spectroscopies. *J. Power Sources* **124**, 518-525 (2003).

3 Otto, S.-K. et al. In-Depth Characterization of Lithium-Metal Surfaces with XPS and ToF-SIMS: Toward Better Understanding of the Passivation Layer. *Chem. Mater.* **33**, 859-867 (2021).

4 Örnek, C., Leygraf, C. & Pan, J. On the Volta potential measured by SKPFM–fundamental and practical aspects with relevance to corrosion science. *Corros. Eng. Sci. Technol.* **54**, 185-198 (2019).

5 Rosenkranz, C. & Janek, J. Determination of local potentials in mixed conductors—two examples. *Solid State Ion.* **82**, 95-106 (1995).

6 Mizusaki, J.-i., Fueki, K. & Mukaibo, T. An Investigation of the Hebb-Wagner's dc Polarization Technique I. Steady-state Chemical Potential Profiles in Solid Electrolytes. *Bull. Chem. Soc. Jpn.* **48**, 428-431 (1975).

7 Liang, C. et al. Unravelling the room-temperature atomic structure and growth kinetics of lithium metal. *Nat. Commun.* **11**, 1-11 (2020).

8 Xia, W. *et al.* Ionic conductivity and air stability of Al-doped $Li_7La_3Zr_2O_{12}$ sintered in alumina and Pt crucibles. *ACS Appl. Mater. Interfaces* **8**, 5335-5342 (2016).

9 Yang, Y.-N., Li, Y.-X., Li, Y.-Q. & Zhang, T. On-surface lithium donor reaction enables decarbonated lithium garnets and compatible interfaces within cathodes. *Nat. Commun.* **11**, 1-10 (2020).

10 Axt, A., Hermes, I. M., Bergmann, V. W., Tausendpfund, N. & Weber, S. A. Know your full potential: Quantitative Kelvin probe force microscopy on nanoscale electrical devices. *Beilstein J. Nanotechnol.* **9**, 1809-1819 (2018).

11 Sugawara, Y., Miyazaki, M. & Li, Y. J. Surface potential measurement by heterodyne frequency modulation Kelvin probe force microscopy in MHz range. *J. Phys. Commun.* **4**, 075015 (2020).

12 Coffey, D. C. & Ginger, D. S. Time-resolved electrostatic force microscopy of polymer solar cells. *Nat. Mater.* **5**, 735-740 (2006).

13 Colchero, J., Gil, A. & Baró, A. Resolution enhancement and improved data interpretation in electrostatic force microscopy. *Phys. Rev. B* **64**, 245403 (2001).

14 Mascaro, A., Miyahara, Y., Enright, T., Dagdeviren, O. E. & Grütter, P. Review of time-resolved non-contact electrostatic force microscopy techniques with applications to ionic transport measurements. *Beilstein J. Nanotechnol.***10**, 617-633 (2019).

15 Kohn, P., Schröter, K. & Thurn-Albrecht, T. Interfacial polarization and field-induced orientation in nanostructured soft-ion conductors. *Phys. Rev. Lett.* **102**, 216101 (2009).

16 Harrison, J. S. et al. Noncontact Imaging of Ion Dynamics in Polymer Electrolytes with Time-Resolved Electrostatic Force Microscopy. *ACS Nano* **13**, 536-543 (2018).
